# Effectiveness of personalized granola tailored to the gut microbiota for improving gut environment and mood states

**DOI:** 10.3389/fmicb.2025.1607918

**Published:** 2025-07-25

**Authors:** Hiroyuki Sasaki, Hirofumi Masutomi, Yohsuke Yamauchi, Katsuyuki Ishihara, Shinji Fukuda

**Affiliations:** ^1^Research & Development Division, Calbee, Inc., Utsunomiya, Japan; ^2^Metagen, Inc., Tsuruoka, Japan; ^3^Institute for Advanced Biosciences Keio University, Tsuruoka, Japan; ^4^Gut Environmental Design Group, Kanagawa Institute of Industrial Science and Technology, Kawasaki, Japan; ^5^Transborder Medical Research Center, University of Tsukuba, Tsukuba, Japan; ^6^Innovative Microbiome Therapy Research Center, Juntendo University Graduate School of Medicine, Bunkyō, Japan

**Keywords:** precision nutrition, short-chain fatty acids, gut microbiota, prebiotics, granola

## Abstract

The gut microbiota plays a critical role in host metabolism, immunity, and mental health. Short-chain fatty acids (SCFAs), produced through the gut microbial fermentation of dietary fibers, are essential metabolites influencing host physiology. Previous studies have suggested that dietary interventions impact SCFA production, but individual responses vary owing to gut microbiota composition. This study sought to investigate whether personalized granola, formulated based on an individual’s gut microbiota, enhances SCFA production and improves metabolic and mental health outcomes. A single-arm, single-blind, before-and-after study was conducted on 99 participants. Personalized granola (BodyGranola^®^, Calbee, Inc.) was tailored to the gut microbiota composition of each individual by incorporating three prebiotic ingredients selected from six options. Participants consumed 50 g of granola daily for 8 weeks. Fecal samples were collected at baseline, week 4, and week 8 for gut microbiota and intestinal metabolite analysis via 16S rRNA gene sequencing and gas chromatography–mass spectrometry. Mood and defecation were assessed using the Profile of Mood States Second Edition (POMS2), Athens Insomnia Scale (AIS), and defecation questionnaires. Personalized granola consumption tended to increase SCFAs, including acetic acid and caproic acid. The relative abundance of *Bifidobacterium* also increased. POMS2 assessments indicated improvements in vitality and total mood disturbance scores. Stool volume increased, but bloating and gas accumulation worsened. Microbiota-type-specific variations in metabolite production were observed. Personalized granola enhances SCFA production and improves mood, suggesting that dietary interventions tailored to gut microbiota composition may optimize health outcomes. Future studies should explore gut microbiota-based precision nutrition in larger, controlled trials.

## 1 Introduction

Approximately 40 trillion gut bacteria reside in the intestines of mammals, collectively referred to as the gut microbiota. In recent years, research has demonstrated that the gut microbiota plays a crucial role in various physiological processes, including host immunity, nutrient absorption, and metabolism (Marchesi et al., 2016). Among the metabolites produced by the gut microbiota, short-chain fatty acids (SCFAs) play a critical role in regulating host physiological functions. SCFAs contribute to a range of processes, such as intestinal immunity, metabolic regulation, endocrine signaling, and neural function. A reduction in SCFA production has been associated with the development of several diseases, including obesity, hypertension, and central nervous system disorders (Chambers et al., 2018; Silva et al., 2020; Overby and Ferguson, 2021; Lange et al., 2023).

SCFAs are produced when the gut microbiota ferments and breaks down indigestible dietary components, such as dietary fiber and resistant oligosaccharides (Koh et al., 2016). When dietary fiber reaches the large intestine, it is hydrolyzed into monosaccharides by gut bacteria-specific enzymes. Most of these monosaccharides are subsequently converted into pyruvate via the glycolytic pathway, and SCFAs are subsequently synthesized from pyruvate through distinct microbial metabolic pathways (Koh et al., 2016). As is evident from this process, indigestible nutrients play a critical role in SCFA production. In particular, prebiotics—indigestible compounds that selectively stimulate beneficial gut bacteria—have been shown to significantly influence gut microbiota composition and SCFA production (David et al., 2014). For instance, inulin, a well-characterized prebiotic, has been reported to alter gut microbiota composition and promote the growth of butyrate-producing bacteria, which generate a key SCFA, butyrate (Valcheva et al., 2015; Barber et al., 2022). Moreover, reduced SCFA levels have been implicated in the development of major depressive disorder and schizophrenia (Yu et al., 2024). Animal studies have demonstrated that butyrate administration can alleviate depressive-like behavior and manic states in models of depression and bipolar disorder (Resende et al., 2013; Wei et al., 2014).

Granola is a food made from a mixture of oats, rye, and other grains and is rich in dietary fiber (Yamauchi et al., 2023). Owing to its high fiber content, granola is believed to influence gut microbiota composition. In fact, studies have reported that consuming granola for breakfast promotes bowel movements in both adult women and elementary school children (Kyo et al., 2017; Matsumoto et al., 2023). Furthermore, in patients undergoing hemodialysis, granola consumption has been shown to alter gut microbiota composition, reduce blood pressure, and lower the serum levels of the uremic toxin indoxyl sulfate (Nagasawa et al., 2021; Nagasawa et al., 2024). Recent studies have reported that consuming granola containing multiple prebiotic ingredients can alter the gut microbiota, including an increase in *Bifidobacterium*. These changes have been associated with reduced perceived stress, improved mood, and a decrease in subjective feelings of sleepiness (Sasaki et al., 2025). Based on the above findings, granola consumption has the potential to promote SCFA production. However, when we measured SCFA levels and various other metabolites in individuals who consumed granola, we found no significant increase in SCFA levels (Yamauchi et al., 2023). One possible explanation for this lack of increase is the presence of substantial interindividual variability—while some individuals exhibited increased SCFA production, others did not. However, a positive correlation was observed between changes in SCFA levels and the abundance of *Prevotella 9*. In other words, the greater the abundance of *Prevotella 9* in the gut, the more granola consumption-promoted SCFA production was observed (Yamauchi et al., 2023). Individuals who exhibit a response to dietary interventions are referred to as responders, while those who do not are classified as non-responders. It is believed that these differences between responders and non-responders are primarily determined by an individual’s gut microbiota composition (Korpela et al., 2014).

Previous studies have shown that individuals can be classified as responders or non-responders in response to prebiotics. For example, barley consumption has been associated with improved glucose metabolism and enhanced blood glucose regulation. However, this effect is primarily observed in responders—individuals with a high abundance of *Prevotella* in their gut microbiota exhibit greater improvements in glucose metabolism (Kovatcheva-Datchary et al., 2015). In addition, studies have identified distinct groups based on their gut microbiota response to inulin consumption. One group exhibited significant changes in microbiota composition, while another exhibited no substantial increase in SCFA levels. Notably, the former group tended to consume a high-fiber diet regularly and harbored a greater abundance of *Bifidobacterium* in the gut (Healey et al., 2018).

As described above, the composition of the gut microbiota varies among individuals, making it challenging to identify a single diet that is optimal for everyone. In recent years, the importance of personalized and precision nutrition—approaches that integrate gut microbiota data to tailor dietary recommendations for individuals—has been increasingly recognized (Kolodziejczyk et al., 2019; Agrawal et al., 2024). Additionally, a narrative review reported that personalizing dietary interventions based on factors such as gut microbiota, metabolic profiles, and genetic variations may help improve risk factors for obesity and type 2 diabetes (Antwi, 2023). Therefore, in the case of prebiotics, it is considered important to account for an individual’s gut microbiota composition and provide prebiotics tailored to enhance the production of metabolites, including SCFAs. However, only a few studies have implemented interventions involving the administration of prebiotics tailored to an individual’s gut microbiota. Therefore, based on previous research, we identified gut bacteria that actively metabolize specific prebiotics and promote SCFA production. Using this information, we developed personalized granola designed to match an individual’s gut microbiota by optimizing the combination of prebiotics and gut bacteria. The aim of this study was to evaluate the effectiveness of personalized granola, hypothesizing that it would significantly enhance the production of metabolites, including SCFAs. In addition to metabolite production, we also aimed to evaluate whether personalized granola improves stool frequency and mental health indicators such as stress levels, given their reported associations with gut microbiota and SCFA levels.

## 2 Materials and methods

### 2.1 Formulation of personalized granola tailored to the gut microbiota

In this study, we developed personalized granola (BodyGranola^®^, Calbee, Inc., Tokyo, Japan) tailored to each individual’s gut microbiota using the following method. Based on a 640 g batch of granola, three out of six prebiotic toppings were selected according to the individual’s gut microbiota composition. Each selected topping (120 g) was added to the base granola and thoroughly mixed, with daily consumption set at 50 g. The six prebiotic toppings included inulin, barley, fructo-oligosaccharide, resistant starch, galacto-oligosaccharide, and Hi-Cacao (high-cacao chocolate). Drawing from previous research, we identified gut bacterial species that actively metabolize each prebiotic and contribute to SCFA production. The combinations of prebiotic toppings and their corresponding gut bacterial species are presented in Table 1. For the personalization process, we focused on six specific gut bacterial genera. After measuring each participant’s gut microbiota composition, we identified the top three genera (by relative abundance) among the six, and selected the corresponding prebiotic toppings to create a customized granola formulation. The matching between prebiotics and bacterial genera was based on literature reports that linked specific prebiotics to the growth or metabolic activation of particular gut bacteria involved in SCFA production. This strategy was designed to target the most dominant genera in each individual’s gut to potentially maximize metabolic response. However, we acknowledge that this genus–prebiotic matching was based on indirect evidence, and no direct functional validation was performed in this study. The nutritional composition of the base granola and prebiotic toppings is provided in Table 2, while the ingredient details for each granola formulation and prebiotic topping are listed in Supplementary Table 1. We selected the top three genera (based on relative abundance) out of six candidate genera to simplify the formulation process and ensure palatability, as the number of prebiotic toppings was limited to three per granola batch. This approach was also intended to maximize the potential response by targeting the most dominant bacterial genera. However, we acknowledge that this selection criterion was somewhat arbitrary and have discussed its limitations and the need for refinement in the Discussion section.

**Table 1:** Combination of prebiotic-toppings and corresponding gut bacteria.

Prebiotic-toppings	Gut bacteria	Main metabolites	References
Inulin	*Bacteroides*	Propionic acid	Aoki et al. (2021) and Chambers et al. (2019)
Barley	*Prevotella*	Succinic acid	Kovatcheva-Datchary et al. (2015) and Fehlbaum et al. (2018)
FOS	*Faecalibacterium*	Butyric acid	Tochio et al. (2018) and Scott et al. (2014)
RS	*Ruminococcus*	Acetic acid	Ze et al. (2012) and Abell et al. (2008)
GOS	*Bifidobacterium*	Acetic acid	Liu et al. (2017) and Fehlbaum et al. (2018)
Hi-Cacao	*Blautia*	Acetic acid	Shin et al. (2022) and Tzounis et al. (2008)

FOS, fructooligosaccharide; RS, resistant starch; GOS, galactooligosaccharide; Hi-Cacao, high cacao chocolate.

**Table 2:** Nutritional composition of fruit granola and each prebiotic-toppings (/100 g).

Nutritional component	Fruit granola	Inulin	Barley	FOS	RS	GOS	Hi-Cacao
Energy (kcal)	443.8	366.7	383.3	366.7	416.7	350	433.3
Protein (g)	6.9	6.7	6.7	6.7	5	6.7	10
Fat (g)	15.9	5	5	5	11.7	3.3	11.7
Carbohydrate (g)	72.5	83.4	83.3	83.3	80	86.6	73.4
Sugar (g)	63.4	61.7	73.3	60	66.7	63.3	66.7
Fiber (g)	9.1	21.7	10	23.3	13.3	23.3	6.7
Sodium content (g)	0.6	0.5	0.5	0.7	0.7	0.3	0.7

FOS, fructooligosaccharide; RS, resistant starch; GOS, galactooligosaccharide; Hi-Cacao, high cacao chocolate.

### 2.2 Participants

**Figure 1:**
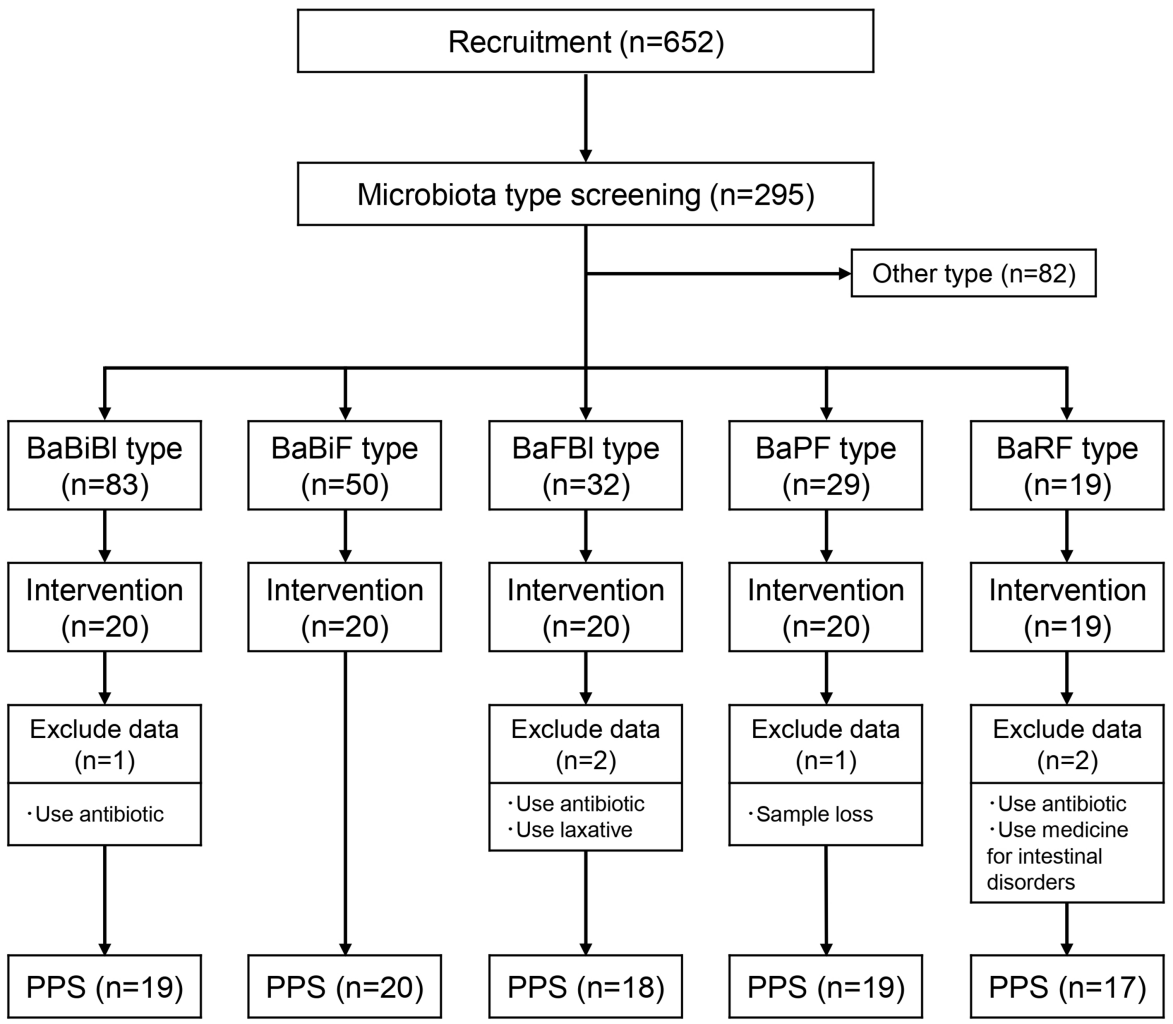
Flow diagram of this trial. Number of participants classified into each gut microbiota type and number of participants who underwent the intervention. PPS, per protocol set; BaBiBl type, gut microbiota type with a high relative abundance of *Bacteroides*, *Bifidobacterium*, and *Blautia*; BaBiF type, gut microbiota type with a high relative abundance of *Bacteroides*, *Bifidobacterium*, and *Faecalibacterium*; BaFBl type, gut microbiota type with a high relative abundance of *Bacteroides*, *Faecalibacterium*, and *Blautia*; BaPF type, gut microbiota type with a high relative abundance of *Bacteroides*, *Prevotella*, and *Faecalibacterium*; BaRF type, gut microbiota type with a high relative abundance of *Bacteroides*, *Ruminococcus*, and *Faecalibacterium*.

The study flow, from participant selection to intervention and analysis, is illustrated in Figure 1. Participants were recruited through the physical management app “Calomama,” and a total of 652 men and women aged 20–65 years were enrolled. The inclusion criteria were as follows: (1) men and women aged between 20 and 65 years at the time of consent; (2) registered users of the “Calomama” app; (3) individuals with a habit of eating breakfast and who were able to replace their regular breakfast with the test food during the intervention period; (4) individuals capable of collecting a fecal sample at home and mailing it to a designated laboratory; and (5) individuals who received a full explanation of the study, understood its content, and provided written informed consent. From this pool, 300 participants were selected based on predefined exclusion criteria. The exclusion criteria were as follows: (1) individuals who had taken or planned to take laxatives, antibiotics, or intestinal medications within 1 month prior to the start of the study; (2) individuals who had undergone an appendectomy; (3) individuals who had undergone surgery within the past 6 months that could potentially influence the study results (e.g., colonoscopy, gallbladder removal, gallstone removal, gastric bypass surgery); (4) individuals currently participating in, or planning to participate in, a clinical trial for another pharmaceutical product or health food; (5) individuals with highly irregular dietary habits; (6) heavy drinkers; (7) individuals living with another study participant; (8) individuals whose lifestyle would change significantly during the trial period, such as shift workers or those being transferred; (9) individuals unable to consume milk; (10) individuals with a history of serious heart, liver, kidney, gastrointestinal (including constipation, diarrhea, inflammatory bowel disease, or colitis), or other diseases, or those currently diagnosed with such conditions; (11) pregnant, breastfeeding, or those planning to become pregnant; (12) individuals with lactose intolerance; and (13) individuals with food allergies. These conditions were assessed using a self-administered pre-screening questionnaire, which asked participants to report any history or current treatment for gastrointestinal or systemic diseases. In addition, participants reported their typical defecation frequency and stool consistency, as part of the baseline data collection.

Next, a fecal collection kit (Mykinso, Cykinso, Inc., Tokyo, Japan) was mailed to the 300 selected participants, and their gut microbiota was analyzed to classify microbiota types. Based on previous research (Watanabe et al., 2021; Yoshida et al., 2022), and the method used to select the three gut bacterial species as mentioned earlier, it has been established that 80% of the Japanese population can be categorized into the following five gut microbiota types: (1) BaBiBl type: high relative abundance of *Bacteroides*, *Bifidobacterium*, and *Blautia*; (2) BaBiF type: high relative abundance of *Bacteroides*, *Bifidobacterium*, and *Faecalibacterium*; (3) BaFBl type: high relative abundance of *Bacteroides*, *Faecalibacterium*, and *Blautia*; (4) BaPF type: high relative abundance of *Bacteroides*, *Prevotella*, and *Faecalibacterium*; and (5) BaRF type: high relative abundance of *Bacteroides*, *Ruminococcus*, and *Faecalibacterium*. Among the 295 participants who returned their fecal samples, the distribution of gut microbiota types was as follows: 83 participants had the BaBiBl type; 50 had the BaBiF type; 32, the BaFBl type; 29, the BaPF type; 19, the BaRF type; and 82 belonged to other microbiota types. The following participants were excluded from the five gut microbiota types, and 20 individuals from each type were selected to receive the intervention according to the following groups: (1) individuals who consume cereal at least 3 days per week; (2) individuals who consume cereal at least 5 days per week; (3) individuals who eat mushrooms at least five-to-six times per week; (4) individuals who consume oligosaccharides at least five-to-six times per week; (5) individuals who drink lactic acid bacteria beverages or take probiotic supplements daily; (6) individuals who consume kimchi or natto at least 5 days per week; and (7) individuals who take Chinese herbal medicine or vitamin supplements daily. The physical characteristics of the selected participants are presented in Table 3.

**Table 3:** Physical characteristics.

Type	Number of subjects		Age	Height	Weight
	Male	Female			
BaBiBl	8	11	43.00 ± 2.28	164.7 ± 2.46	63.4 ± 4.56
BaBiF	6	14	48.05 ± 1.69	163.5 ± 1.72	61.4 ± 2.24
BaFBl	6	12	47.83 ± 2.39	163.0 ± 1.66	60.4 ± 2.67
BaPF	6	13	44.35 ± 1.81	163.8 ± 1.77	62.3 ± 2.84
BaRF	6	11	52.88 ± 1.81	160.9 ± 1.52	57.8 ± 2.32
Total	32	61	47.71 ± 0.94	163.3 ± 0.83	61.2 ± 1.36

Values are means ± SEM.

This study was conducted in accordance with the principles of the Declaration of Helsinki and was approved by the Ethics Review Committee of the Chiyoda Paramedical Care Clinic (Approval Number: 15000088). Informed consent was obtained from all participants prior to enrollment. This clinical trial is registered with the institutional ethics committee of the Japanese University Hospital Medical Information Network (Clinical Trial Reference Number: UMIN000053204).

### 2.3 Study design and procedure

**Figure 2:**
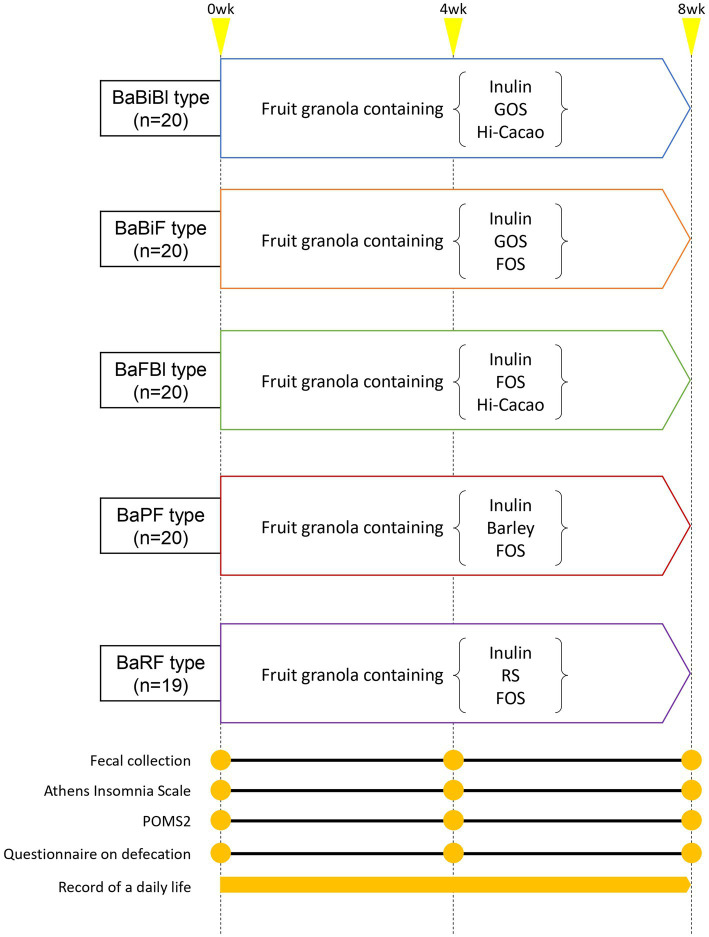
Intervention diagram. Participants consumed personalized granola (fruit granola with three prebiotic toppings) for 8 weeks, depending on their gut microbiota type. Questionnaires and fecal samples were collected at weeks 0, 4 and, 8 (yellow circle points). A daily life diary was recorded throughout the study period.

The study was conducted between December 2023 and June 2024 as a single-arm, non-targeted, single-blind, before-and-after comparison study. All 99 participants were instructed to consume 50 g of personalized granola (test meal) with 200 mL of milk for breakfast daily for 8 weeks and record their lifestyle data in the “Calomama” app. Participants collected fecal samples using a fecal collection kit at the following time points: week 0 (from 4 days before to 1 day before consuming the test meal), week 4 (from day 24 to day 28 after starting test meal consumption), and week 8 (from day 52 to day 56 after starting test meal consumption). The samples were then mailed for analysis. Additionally, participants completed a questionnaire on defecation status, which included the following items: (1) defecation frequency (number of bowel movements per week); (2) stool volume, assessed using an egg-based equivalency scale; and (3) stool consistency, evaluated using the Bristol Stool Scale (BSS). Furthermore, participants responded to the Athens Insomnia Scale (AIS) and Profile of Mood States 2 (POMS2) questionnaire (Figure 2). During the study period, 93 participants were included in the per protocol set (PPS) analysis, after excluding five participants who used antibiotics, laxatives, or intestinal medications, and one participant who lost their sample. Although participants’ diets were not strictly controlled, they were instructed to maintain their habitual dietary patterns and avoid major dietary changes during the study period, particularly regarding alcohol and probiotic or prebiotic supplement intake. Dietary intake was monitored using the “Calomama” app, which allows users to log their daily meals. However, not all participants recorded their meals consistently, and the calculated values for energy and nutrient intake were found to be unreliable due to underreporting and input variability. Therefore, precise dietary intake data could not be included in the analysis. Nevertheless, the consumption of fermented foods commonly found in the Japanese diet, such as natto, was assessed through a pre-study questionnaire. This information was used as part of the participant selection and screening process. The AIS, POMS2, and questionnaire on defecation were self-administered and completed individually at home, without the influence of study personnel. To enhance neutrality and reduce potential bias, a third-party organization (CPCC Co., Ltd.) was contracted to manage conducting test, the participants and the distribution, collection, and aggregation of questionnaire data. Participants were informed that their responses would be treated confidentially and used solely for research purposes, which helped minimize social desirability bias. Furthermore, the use of validated and widely adopted instruments, such as the AIS and POMS2 (Soldatos et al., 2000, 2003; McNair et al., 2012), supports the robustness of the data.

### 2.4 Organic acid level measurements

Metabolites were extracted from fecal samples collected using MG kit (Metagen, Inc. Japan) as previously described (Hashimoto et al., 2023). Dried fecal samples were mechanically disrupted by shaking with 3.0 mm zirconia beads at 1,500 × g for 10 min using a Shake Master (Biomedical Science, Tokyo, Japan). A 10 mg fecal sample was suspended in 100 μL of purified water containing 500 μM crotonic acid as an internal standard. Thereafter, 50 μL of hydrochloric acid and 200 μL of diethyl ether were added. The mixture was shaken at 1,500 × g for 10 min using the Shake Master and then centrifuged at 10,000 × g for 10 min. After centrifugation, 80 μL of the upper ether layer was collected, and 16 μL of derivatization reagent (MTBSTFA: N-tert-butyldimethylsilyl N-methyltrifluoroacetamide) was added. The mixture was transferred to an Agilent crimp-cap vial, which was sealed and incubated at 80°C for 20 min. The sample was then left at room temperature overnight for derivatization. A 2 μL aliquot of the derivatized sample was injected into a 7,890 series GC-MS system (Agilent Technologies, CA, United States) equipped with a DB-5 ms column (0.25 mm × 30 m × 0.25 μm, Agilent Technologies). Helium was used as the carrier gas at a flow rate of 1.2 mL/min. The head pressure was set to 10.5 psi with a 100:1 split ratio. The injector, ion source, quadrupole mass analyzer, and transfer line temperatures were set to 250°C, 230°C, 150°C, and 260°C, respectively. Measurements were conducted using the Agilent MassHunter Workstation Data Acquisition software (version 10.0, Agilent Technologies), and data were analyzed using the Agilent MassHunter Quantitative Analysis software (version 10.1, Agilent Technologies).

### 2.5 DNA extraction from fecal sample

Fecal samples were collected using a fecal collection kit containing guanidine thiocyanate solution. The collected samples were shipped at room temperature and stored at 4°C upon arrival. DNA extraction was performed using an automated DNA extractor (GENE PREP STAR PI-480; Kurabo Industries Ltd., Osaka, Japan) according to the manufacturer’s protocol.

### 2.6 16S rRNA gene sequencing and analysis

The V1–V2 region of the 16S rRNA gene was amplified from extracted DNA using the following primers and KAPA HiFi HotStart ReadyMix (Roche, Basel, Switzerland):

Forward primer: 16S 27Fmod = TCG TCG GCA GCG TCA GAT GTG TAT AAG AGA CAG AGR GTT TGA TYM TGG CTC AG.
Reverse primer: 16S 338R = GTC TCG TGG GCT CGG AGA TGT GTA TAA GAG ACA GTG CTG CCT CCC GTA GGA GT.

Library preparation was performed according to the Illumina 16S library preparation protocol (Illumina, San Diego, CA, United States) as follows. Using the Nextera XT Index Kit (Illumina, San Diego, CA, United States), libraries were prepared with dual index adapters. Each library was diluted to 5 ng/μL, and equal amounts were pooled to a final concentration of 4 nM. The DNA concentration of the libraries was quantified by qPCR using KAPA SYBR FAST qPCR Master Mix (KK4601, KAPA Biosystems).

Sequencing was performed using the MiSeq Reagent Kit V2 (500 cycles), generating 250 bp paired-end reads. Paired-end reads were analyzed for gut microbiota composition using QIIME2 (version 2024.2) (Bolyen et al., 2019). First, DADA2 was applied to denoise and low-quality reads were filtered, generating amplicon sequence variants (ASVs) (Callahan et al., 2016). Taxonomic classification of ASVs was performed using the Silva SSU Ref Nr 99 (version 132) classifier (Quast et al., 2013). Additionally, alpha diversity was calculated for the ASVs using three indices: Chao1, Shannon, and Simpson.

### 2.7 AIS

Sleep disturbances, particularly insomnia, were assessed using the AIS. The AIS is a questionnaire consisting of eight items (Soldatos et al., 2000, 2003), each rated on a four-point scale: (0 = no problem at all, 1 = slight problem, 2 = significant problem, 3 = very significant problem). The questionnaire evaluates the following aspects of sleep: (1) difficulty falling asleep, (2) nighttime awakenings, (3) early morning awakenings, (4) total sleep duration, (5) overall sleep quality, (6) daytime mood state, (7) daytime mental and physical functioning, and (8) daytime sleepiness. The total score of these eight items constitutes the AIS score. A cutoff score >6 is used to identify insomnia (Soldatos et al., 2003).

### 2.8 POMS2

The Japanese version of POMS2 was used to assess mood states (McNair et al., 2012). The questionnaire consists of seven mood scales: (1) anger–hostility (AH), (2) confusion–bewilderment (CB), (3) depression–dejection (DD), (4) fatigue–inertia (FI), (5) tension–anxiety (TA), (6) vigor–activity (VA), and (7) friendship (F). Additionally, the total mood disturbance (TMD) score was calculated as an overall measure of mood state. To reduce participant burden, a shortened version of POMS2 was used in this study. Participants completed 35 questions, rating their current emotional state on a 5-point scale from “not at all” (0 points) to “very much” (4 points). The TMD score was calculated by summing the total scores of AH, CB, DD, FI, and TA, and then subtracting the total score of VA. The POMS2 raw scores were converted into standardized T-scores, which were used for mood state evaluation.

### 2.9 Questionnaire on defecation

The following six items were assessed in relation to defecation:

1. Defecation frequency: evaluated based on the number of bowel movements per day.
2. Stool volume: assessed using an equivalency scale based on the number of chicken egg-sized stool units.
3. Stool consistency: rated on a 7-point scale [1 = very hard, 2 = hard, 3 = somewhat hard, 4 = normal, 5 = somewhat soft, 6 = soft (muddy), 7 = very soft (watery)].
4. Feeling of incomplete evacuation: rated on a 4-point scale (1 = persistent discomfort after defecation; 2 = slight feeling of incomplete evacuation; 3 = almost no feeling of incomplete evacuation; somewhat refreshed; and 4 = no feeling of incomplete evacuation; completely refreshed).
5. Bloating severity: rated on a 6-point scale (1 = very strong, 2 = strong, 3 = normal, 4 = weak, 5 = very weak, 6 = not particularly strong).
6. Gas build-up severity: rated on a 6-point scale (1 = very strong, 2 = strong, 3 = normal, 4 = weak, 5 = very weak, 6 = not particularly strong).

### 2.10 Statistical analysis

Statistical analyses were conducted using GraphPad Prism (version 9.5.1, GraphPad Software Inc., United States). All data are presented as mean ± SEM. Statistical comparisons were performed between weeks 0, 4, and 8, while comparisons between gut microbiota types were not conducted. For each variable, normality was assessed using the D’Agostino–Pearson test, followed by variance testing using Bartlett’s test. If the data were normally distributed and had equal variances, a one-way repeated measures ANOVA was applied, with Tukey’s multiple comparison test used as a *post-hoc* test. If the data were non-normally distributed or had unequal variances, the Friedman test (a non-parametric alternative) was performed, followed by Dunn’s multiple comparison test for *post-hoc* analysis. For correlation analysis, Spearman’s correlation coefficient was calculated. *p*-values were computed for each test, and *p* < 0.05 was considered statistically significant.

## 3 Results

### 3.1 Personalized granola consumption increased SCFAs

**Figure 3:**
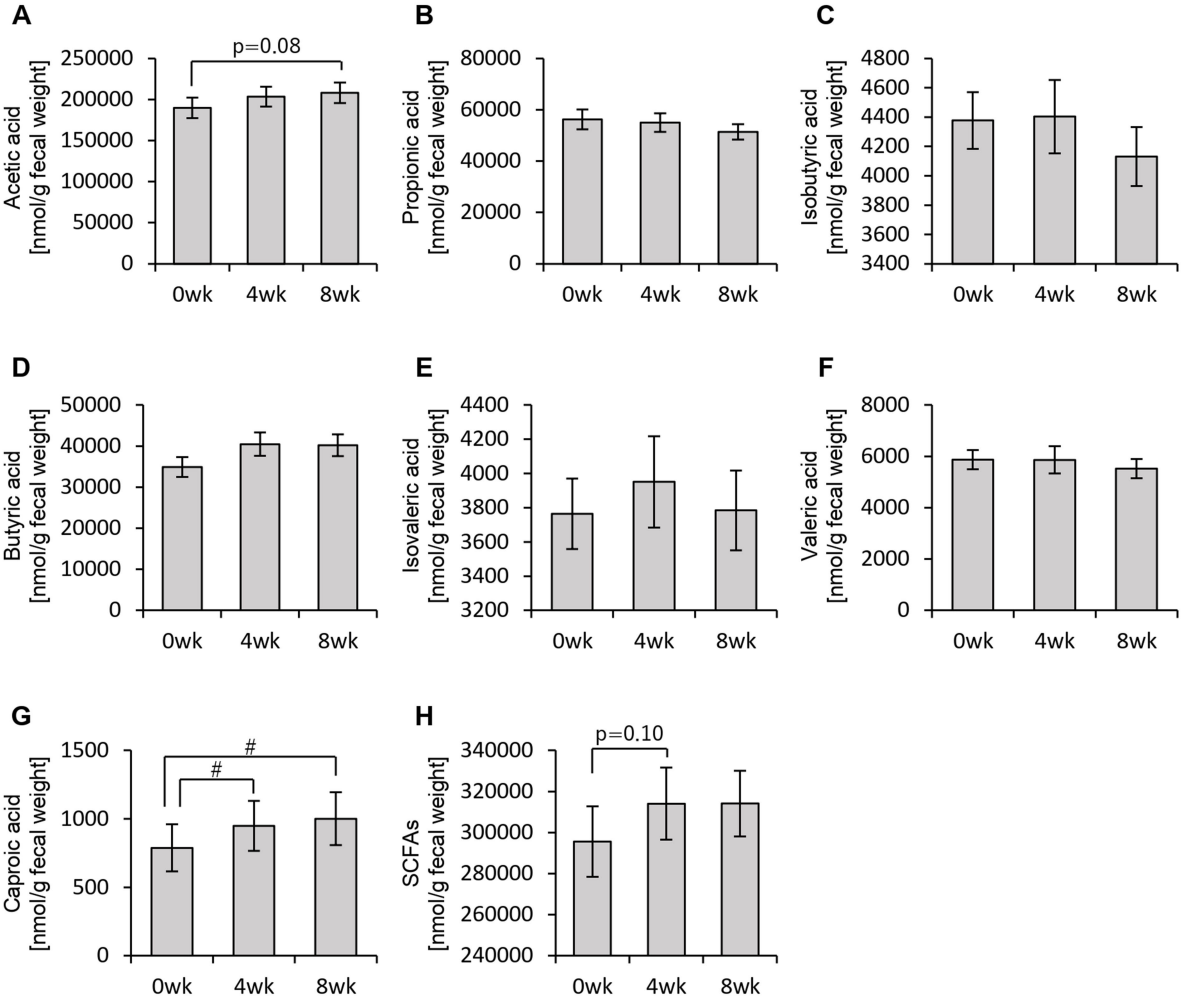
Personalized granola consumption increases short-chain fatty acid production regardless of gut microbiota type. Short-chain fatty acid (SCFA) production in all subjects: **(A)** acetic acid, **(B)** propionic acid, **(C)** isobutyric acid, **(D)** butyric acid, **(E)** isovaleric acid, **(F)** valeric acid, **(G)** caproic acid, and **(H)** SCFAs [the sum of **(A)** through **(G)**]. All values are represented as mean ± SEM (*n* = 93). ^#^*p* < 0.05, evaluated using the Friedman test with Dunn’s *post-hoc* test.

First, we analyzed the effects of consuming personalized granola tailored to each individual’s gut microbiota on the gut environment in 93 participants, examining overall changes regardless of gut microbiota type. Among the organic acids produced as metabolic byproducts, SCFAs such as acetic acid and butyric acid were measured. The results are presented in Figure 3. Acetic acid levels were tended to increase from week 0 to week 8 following the consumption of personalized granola (Figure 3A). Caproic acid levels exhibited significant differences between week 0 and week 4, as well as between week 0 and week 8 (Figure 3G).

**Figure 4:**
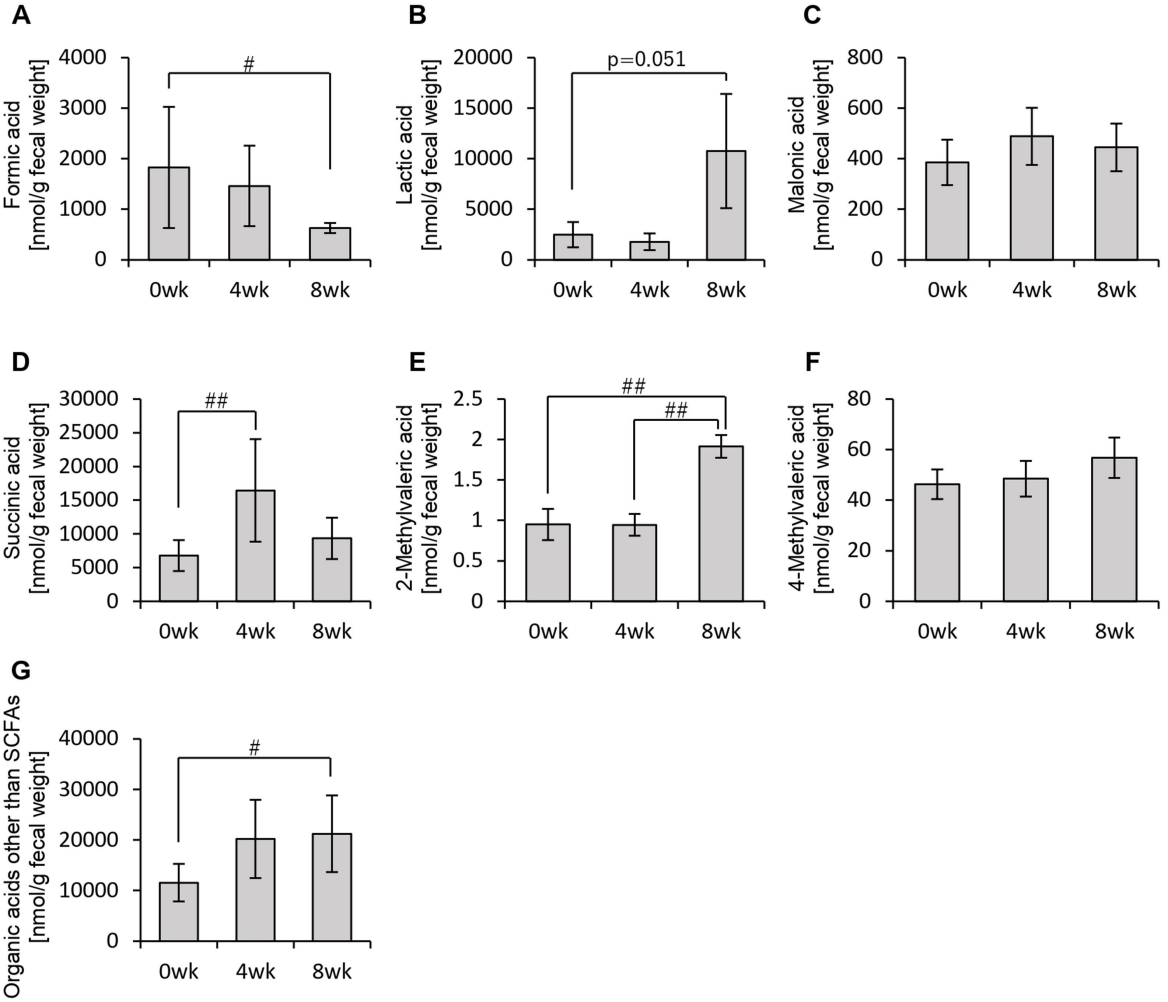
Personalized granola consumption increases organic acids other than SCFA production regardless of gut microbiota type. Organic acid production in all subjects: **(A)** formic acid, **(B)** lactic acid, **(C)** malonic acid, **(D)** succinic acid, **(E)** 2-methylvaleric acid, **(F)** 4-methylvaleric acid, and **(G)** organic acids other than SCFAs [the sum of **(A)** through **(F)**]. All values are represented as mean ± SEM (*n* = 93). ^##^*p* < 0.01 and ^#^*p* < 0.05, evaluated using the Friedman test with Dunn’s *post-hoc* test.

In addition to SCFAs, other organic acids were also analyzed (Figure 4). Formic acid levels decreased significantly from week 0 to week 8 (Figure 4A). Lactic acid levels exhibited an increasing trend from week 0 to week 8, while succinic acid levels increased significantly between week 0 and week 4, week 0 and week 8, as well as between week 4 and week 8 (Figures 4B,D,E). Total organic acid levels, excluding the main SCFAs, increased significantly from week 0 to week 8 (Figure 4G).

These findings indicate that consuming personalized granola leads to an overall increase in organic acids, which are key metabolic substances in the gut, regardless of gut microbiota type.

### 3.2 Relative abundance of *Bifidobacterium* increased with the consumption of personalized granola

The consumption of personalized granola led to an increase in organic acid levels, which are key metabolic substances in the gut. However, we further investigated whether the composition of the gut microbiota responsible for producing these metabolites was altered.

First, we examined overall gut microbiota composition using beta diversity analysis (Supplementary Figure 1 and Supplementary Table 2). The results indicated that while gut microbiota composition varied significantly based on microbiota type, no significant changes were observed within the same microbiota type over the study period. In other words, these findings suggest that consuming personalized granola does not significantly alter the overall gut microbiota composition.

**Figure 5:**
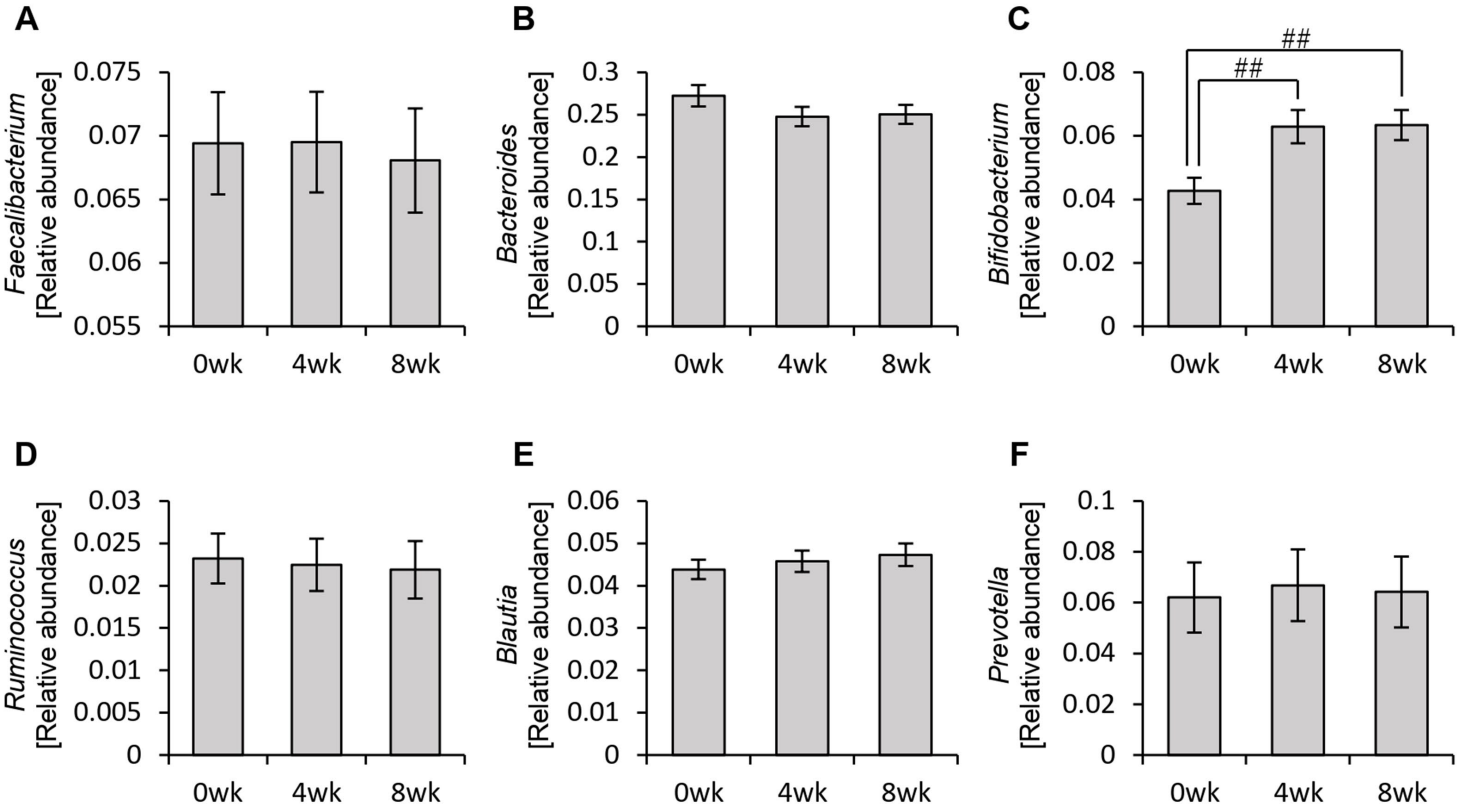
Changes in the six bacterial genera considered when formulating personalized granola. The relative abundance of six bacteria at the genus level in all participants: **(A)** *Faecalibacterium*, **(B)** *Bacteroides*, **(C)** *Bifidobacterium*, **(D)** *Ruminococcus*, **(E)** *Blautia*, and **(F)** *Prevotella*. All values are represented as mean ± SEM (*n* = 93). ^##^*p* < 0.01, evaluated using the Friedman test with Dunn’s *post-hoc* test.

Therefore, rather than analyzing the gut microbiota as a whole, we specifically examined the relative abundance of the six bacterial genera used to define personalized granola (Figure 5). As a result, we observed a significant increase in the relative abundance of *Bifidobacterium* among these six bacterial genera following personalized granola consumption (Figure 5C).

Additionally, analysis of the alpha diversity revealed that the Chao1 and Shannon diversity indices significantly decreased after personalized granola consumption (Supplementary Figure 2).

### 3.3 Personalized granola consumption improved overall mood disturbance

**Figure 6:**
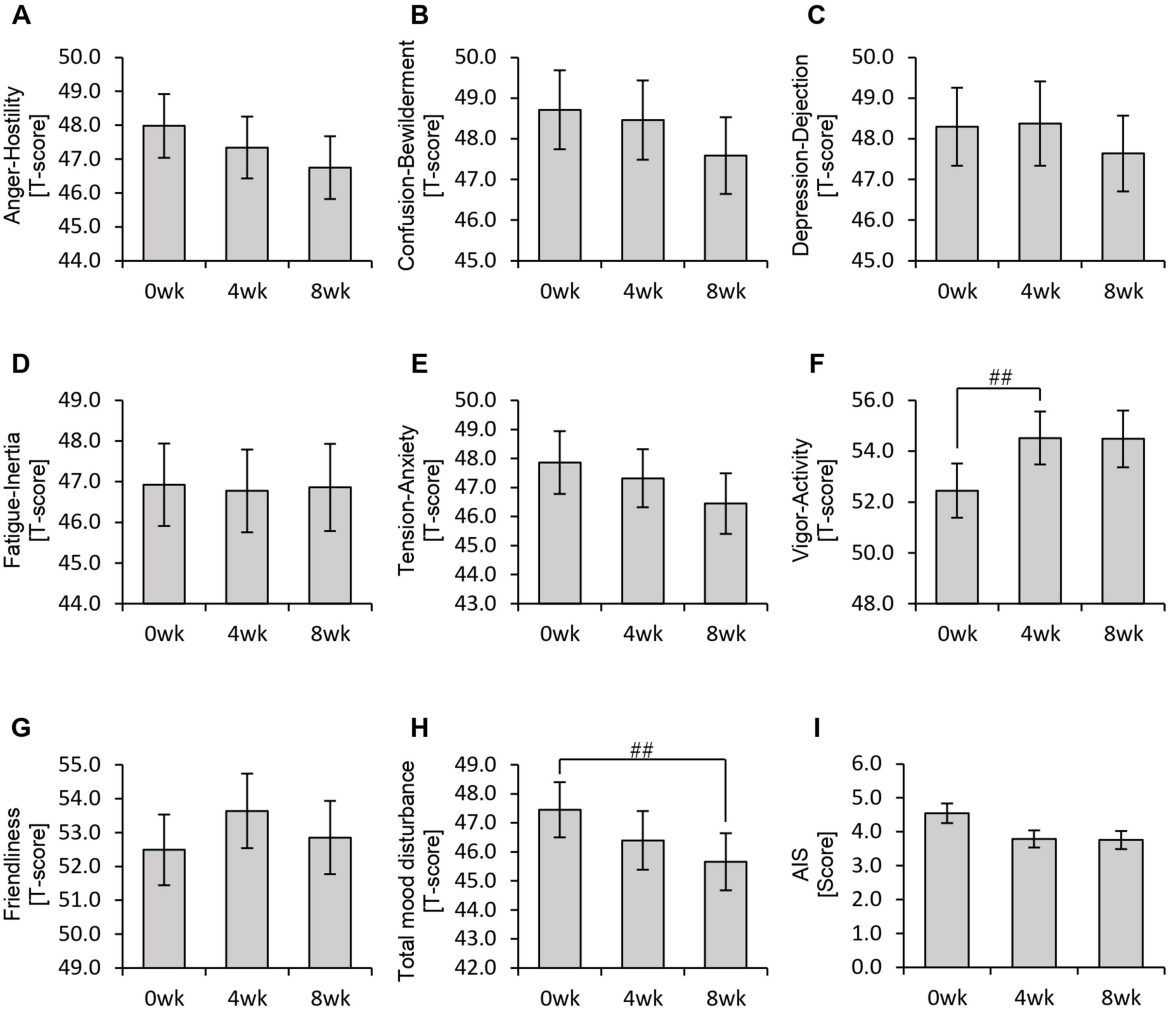
Personalized granola consumption may improve total mood disturbances. Summary of scores obtained from answers to each questionnaire in all participants: **(A–H)** POMS2 [Profile of Mood States 2nd edition **(A)** Anger–Hostility scale, **(B)** Confusion–Bewilderment scale, **(C)** Depression–Dejection scale, **(D)** Fatigue–Inertia scale, **(E)** Tension–Anxiety scale, **(F)** Vigor–Activity scale, **(G)** Friendliness scale, and **(H)** Total mood disturbance]. **(I)** AIS (Athens Insomnia Scale). All values are represented as mean ± SEM (*n* = 93). ^##^*p* < 0.01, evaluated using the Friedman test with Dunn’s *post-hoc* test.

**Figure 7:**
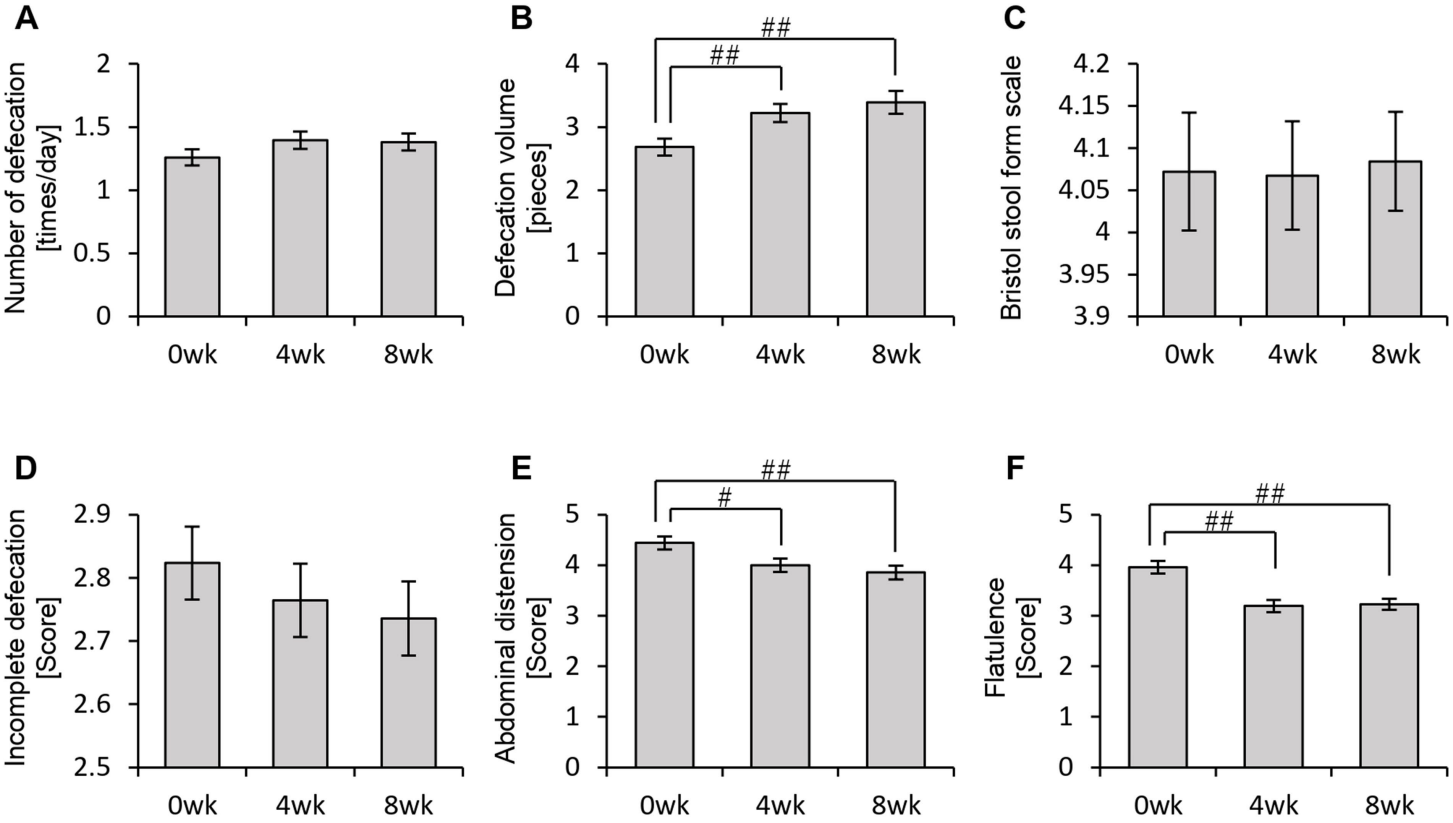
Personalized granola consumption can change defecation conditions. The summary of the results of the questionnaire on defecation condition in all participants: **(A)** number of stools per day; **(B)** defecation volume when converted to the number of eggs; **(C)** Bristol stool form scale; **(D)** incomplete defecation; **(E)** abdominal distension; and **(F)** flatulence. All values are represented as mean ± SEM (*n* = 93). ^##^*p* < 0.01 and ^#^*p* < 0.05, evaluated using the Friedman test with Dunn’s *post-hoc* test.

Thus far, we have confirmed that personalized granola consumption increases metabolite levels, regardless of gut microbiota type, and enhances the relative abundance of *Bifidobacterium*. Considering that gut microbiota-derived metabolites contribute to the regulation of host physiological functions, we hypothesized that this increase in metabolites could influence the host’s mental and physical well-being. To explore this, we analyzed the results of the POMS2, AIS, and defecation questionnaires (Figures 6, 7).

In the POMS2 assessment, a significant improvement was observed in vitality (VA) between week 0 and week 4 (Figure 6F). Additionally, the total TMD score decreased significantly from week 0 to week 8 (Figure 6H), suggesting that consuming personalized granola may contribute to overall mood improvement. However, no significant changes were observed in the AIS (Figure 6I). From the defecation questionnaire, it was found that stool volume increased significantly following personalized granola consumption (Figure 7B). However, the scores for bloating and gas accumulation worsened (Figures 7E,F).

These findings indicate that personalized granola consumption improves mood and increases stool volume, regardless of gut microbiota type. However, it may also lead to side effects including increased bloating and gas accumulation.

### 3.4 Increase in organic acids in different gut microbiota types

We examined the overall effects of personalized granola consumption on the gut microbiota, irrespective of microbiota type. Next, we analyzed the impact of personalized granola consumption on each specific gut microbiota type to determine whether the effects varied by gut microbiota composition.

**Figure 8:**
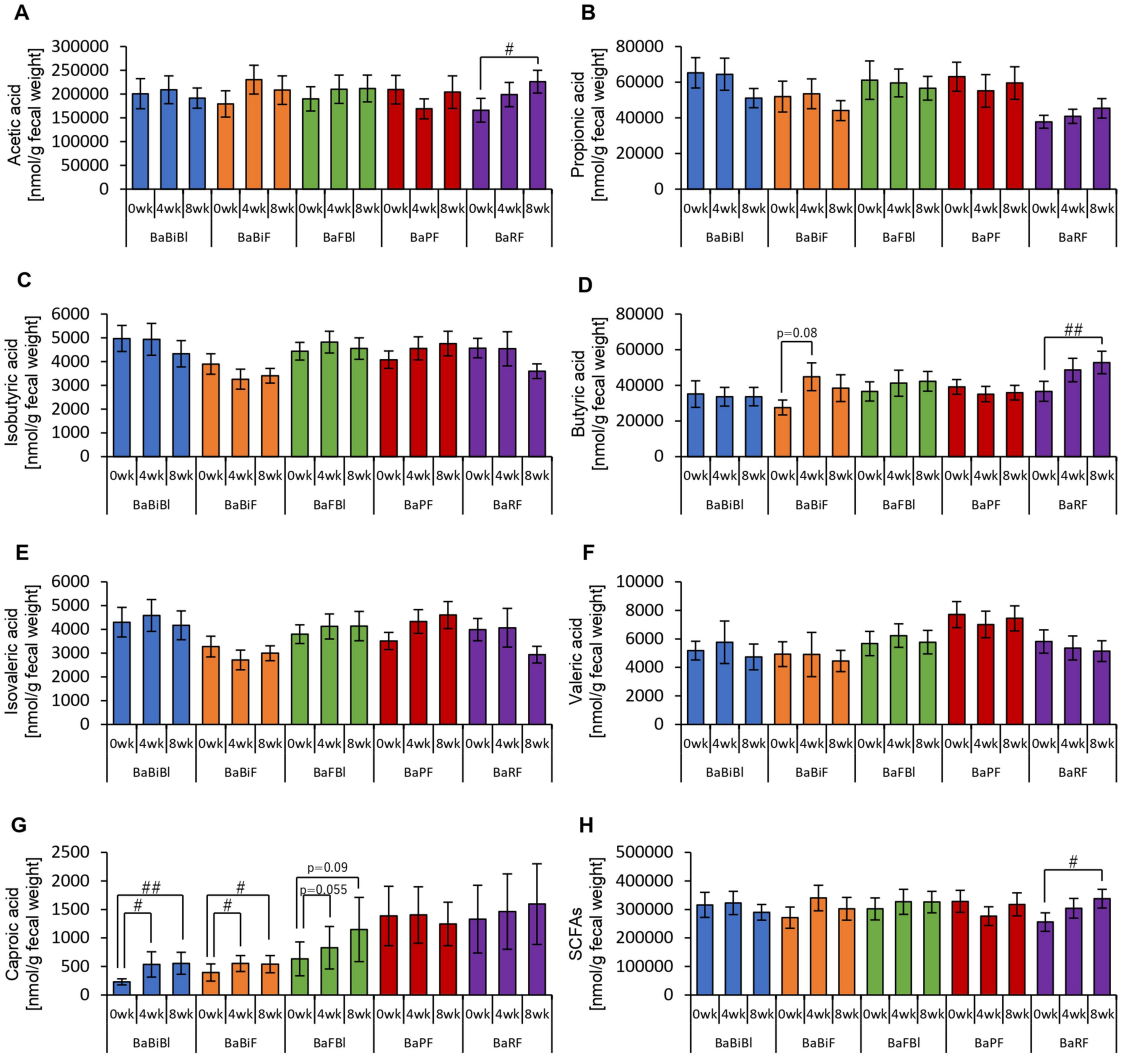
Personalized granola intake increases SCFAs that differ depending on the type of gut microbiota. SCFA production by each type of gut microbiota: **(A)** acetic acid, **(B)** propionic acid, **(C)** isobutyric acid, **(D)** butyric acid, **(E)** isovaleric acid, **(F)** valeric acid, **(G)** caproic acid, and **(H)** SCFAs [the sum of **(A)** through **(G)**]. All values are represented as mean ± SEM (BaBiBl type: *n* = 19, BaBiF type: *n* = 20, BaFBl type: *n* = 18, BaPF type: *n* = 19, and BaRF type: *n* = 17). ^##^*p* < 0.01 and ^#^*p* < 0.05, evaluated using the Friedman test with Dunn’s *post-hoc* test to compare between weeks within the gut microbiota type.

**Figure 9:**
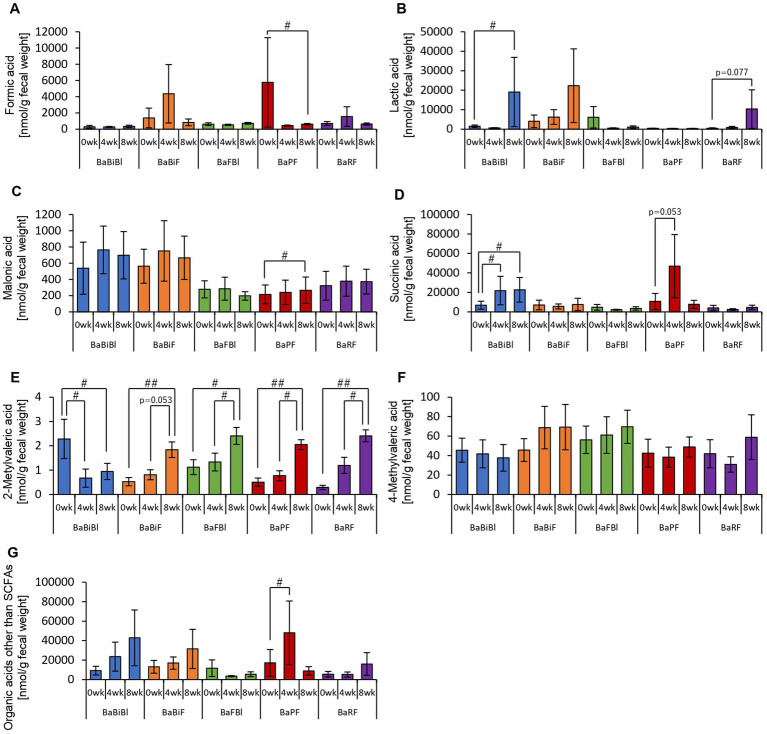
Personalized granola consumption increases organic acids other than SCFAs that differ depending on the type of gut microbiota. Organic acid production based on each type of gut microbiota in all subjects. **(A)** Formic acid, **(B)** lactic acid, **(C)** malonic acid, **(D)** succinic acid, **(E)** 2-methylvaleric acid, **(F)** 4-methylvaleric acid, **(G)** organic acids other than SCFAs [the sum of **(A)** through **(F)**]. All values are represented as mean ± SEM (BaBiBl type: *n* = 19, BaBiF type: *n* = 20, BaFBl type: *n* = 18, BaPF type: *n* = 19, BaRF type: *n* = 17). ^##^*p* < 0.01 and ^#^*p* < 0.05, evaluated using the Friedman test with Dunn’s *post-hoc* test to compare between weeks within the gut microbiota type.

First, we examined the metabolites produced by the gut microbiota in each microbiota type (Figures 8, 9). In the BaBiBl type, caproic acid, lactic acid, and succinic acid increased significantly (Figures 8G, 9B,D), while 2-methylvaleric acid decreased (Figure 9E). In the BaBiF type, butyric acid exhibited an upward trend at week 4 (Figure 8D); in addition, caproic acid and 2-methylvaleric acid increased (Figures 8G, 9E). In the BaFBl type, caproic acid exhibited an increasing trend from week 0 to week 8 (Figure 8G), and 2-methylvaleric acid increased significantly (Figure 9E). In the BaPF type, formic acid decreased significantly from week 0 to week 8 (Figure 9A), while malonic acid and 2-methylvaleric acid increased significantly (Figures 9C,E). Succinic acid also exhibited increasing trend between week 0 and week 4 in this type (Figure 9D). The total amount of organic acids (excluding SCFAs) tended to increase between week 0 and week 4 (Figure 9G). In the BaRF type, acetic acid and butyric acid increased from week 0 to week 8 (Figures 8A,D). While the total SCFA levels increased in this type (Figure 8H), other organic acids also exhibited an increasing trend, with a significant rise in 2-methylvaleric acid levels (Figures 9B,E).

These findings suggest that the types of organic acids produced in response to personalized granola consumption vary depending on gut microbiota type, highlighting distinct metabolic responses across different microbiota compositions.

Next, we examined the relative abundances of the six bacterial genera that were used to define personalized granola.

**Figure 10:**
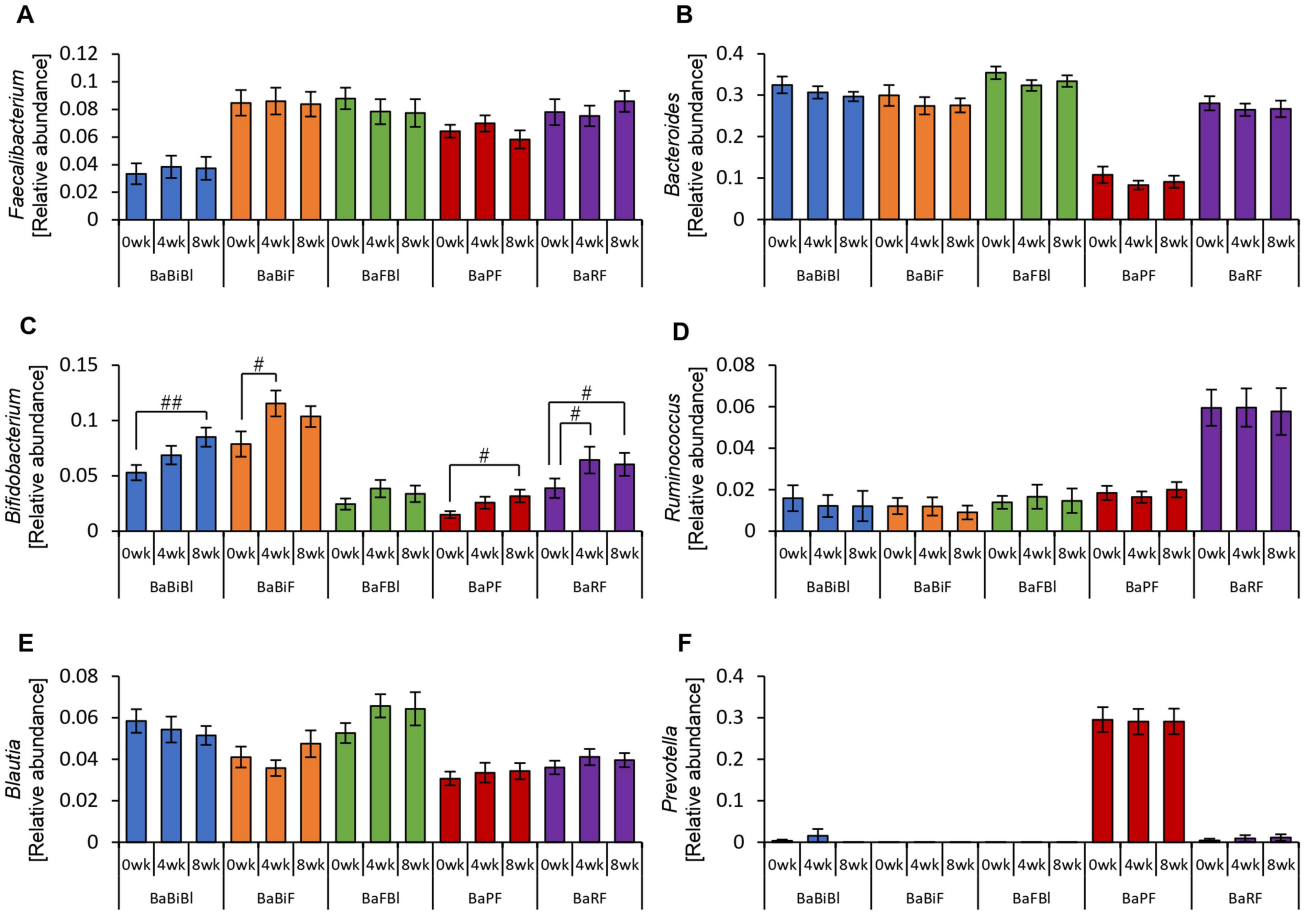
*Bifidobacterium* was altered in most gut microbiota types based on the consumption of personalized granola. The relative abundance of six bacteria at the genus level according to gut microbiota types: **(A)** *Faecalibacterium*, **(B)** *Bacteroides*, **(C)** *Bifidobacterium*, **(D)** *Ruminococcus*, **(E)** *Blautia*, and **(F)** *Prevotella*. All values are represented as mean ± SEM (BaBiBl type: *n* = 19, BaBiF type: *n* = 20, BaFBl type: *n* = 18, BaPF type: *n* = 19, and BaRF type: *n* = 17). ^##^*p* < 0.01 and ^#^*p* < 0.05, evaluated using the Friedman test with Dunn’s *post-hoc* test to compare between weeks within the gut microbiota type.

The relative abundance of *Bifidobacterium* increased between week 0 and week 4 or between week 0 and week 8 in all gut microbiota types except for the BaFBl type (Figure 10C). Notably, *Bifidobacterium* exhibited significant increases, even in microbiota types where it was not among the top three most abundant bacterial species, such as the BaPF and BaRF types. Furthermore, no significant changes were observed in other bacterial genera, such as *Bacteroides* and *Blautia* (Figures 10A,B,D–F).

Analysis of the alpha diversity revealed a significant decrease or a decreasing trend in the Chao1 index across all gut microbiota types (Supplementary Figure 3A).

### 3.5 Disturbances in physical and mental states vary based on gut microbiota type

**Figure 11:**
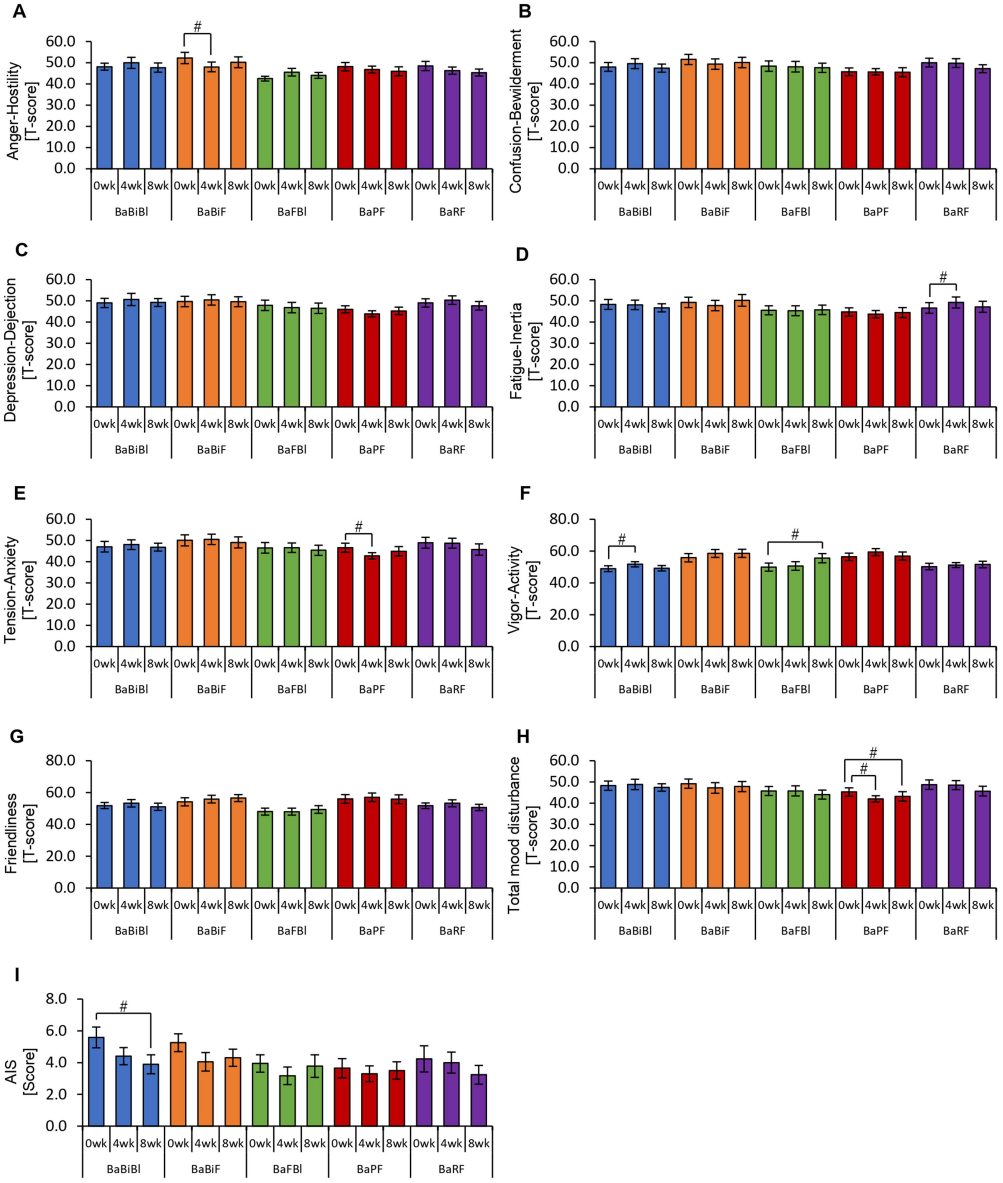
Personalized granola consumption causes changes in mood states that differ depending on the type of gut microbiota. Summary of scores obtained from answers to each questionnaire according to gut microbiota types: **(A–H)** POMS2 [Profile of Mood States 2nd edition **(A)** Anger–Hostility scale, **(B)** Confusion–Bewilderment scale, **(C)** Depression–Dejection scale, **(D)** Fatigue–Inertia scale, **(E)** Tension–Anxiety scale, **(F)** Vigor–Activity scale, **(G)** Friendliness scale, and **(H)** Total mood disturbance]. **(I)** AIS (Athens Insomnia Scale). All values are represented as mean ± SEM (BaBiBl type: *n* = 19, BaBiF type: *n* = 20, BaFBl type: *n* = 18, BaPF type: *n* = 19, and BaRF type: *n* = 17). ^##^*p* < 0.01 and ^#^*p* < 0.05, evaluated using the Friedman test with Dunn’s *post-hoc* test to compare between weeks within the gut microbiota type.

**Figure 12:**
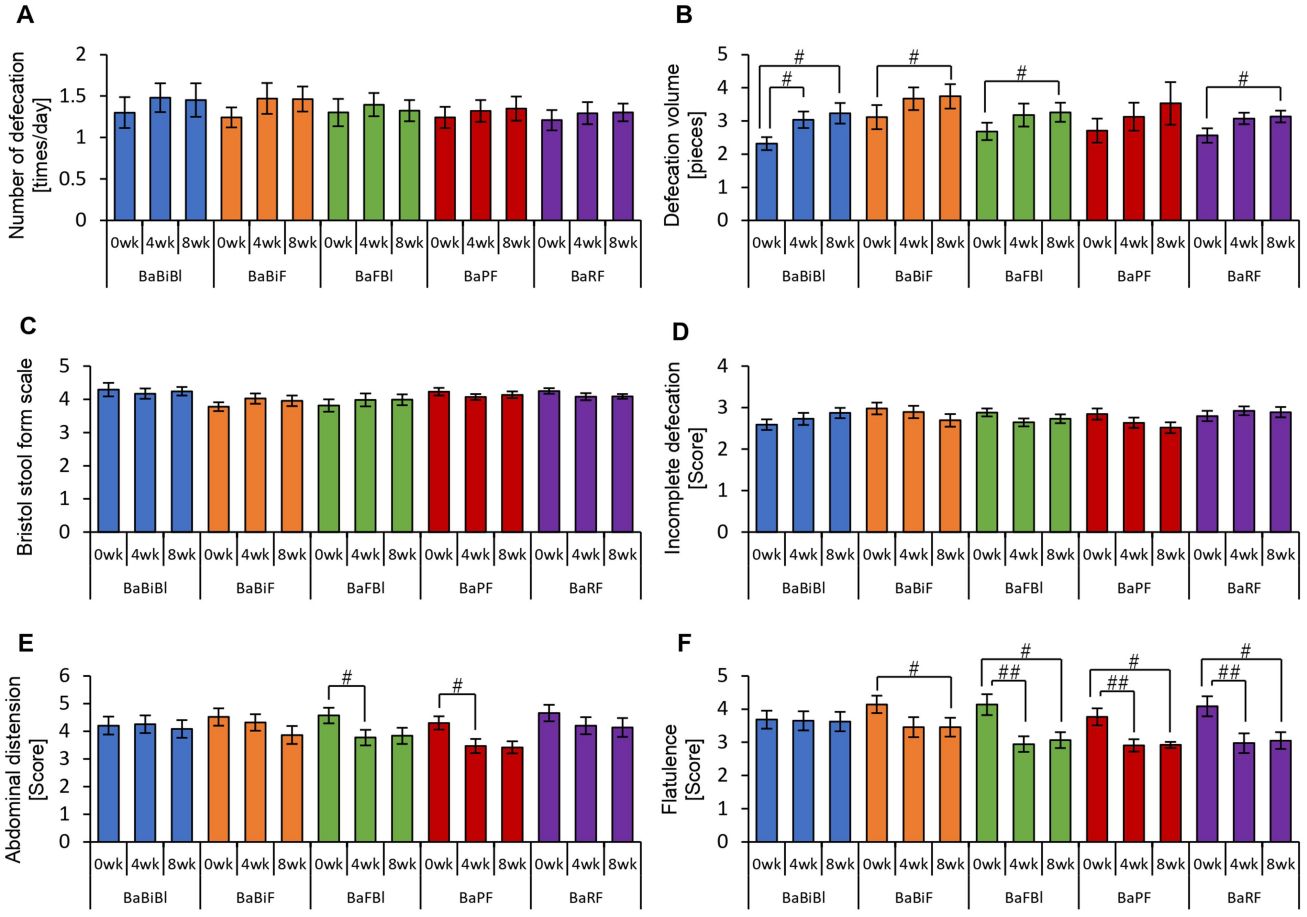
Consumption of personalized granola may increase the defecation volume in any gut microbiota type. The summary of the results of a questionnaire on defecation condition according to gut microbiota types: **(A)** number of stools per day; **(B)** defecation volume when converted to the number of eggs; **(C)** Bristol stool form scale; **(D)** incomplete defecation; **(E)** abdominal distension; and **(F)** flatulence. All values are represented as mean ± SEM (BaBiBl type: *n* = 19, BaBiF type: *n* = 20, BaFBl type: *n* = 18, BaPF type: *n* = 19, and BaRF type: *n* = 17). ^##^*p* < 0.01 and ^#^*p* < 0.05, evaluated using the Friedman test with Dunn’s *post-hoc* test to compare between weeks within the gut microbiota type.

We previously confirmed that different gut microbiota types exhibited distinct metabolic responses to personalized granola consumption. Next, we sought to examine how these variations affected physical and mental states by analyzing data from the POMS2, AIS, and defecation status questionnaires for each microbiota type (Figures 11, 12).

In the BaBiBl type, VA (POMS2) significantly improved from week 0 to week 4 (Figure 11F) while insomnia disturbance (AIS) significantly improved from week 0 to week 8 (Figure 11I). In the BaBiF type, AH (POMS2) significantly improved from week 0 to week 4 (Figure 11A). In the BaFBl type: VA (POMS2) significantly improved from week 0 to week 8 (Figure 11F). In the BaPF type, TA (POMS2) significantly improved from week 0 to week 4, while the TMD scores significantly improved between week 0 and week 4 as well as between week 0 and week 8. In the BaRF type, FI (POMS2) significantly worsened from week 0 to week 4. These findings indicate that specific POMS2 and AIS items were affected differently depending on the gut microbiota type. A significant increase in stool volume was observed in all microbiota types except BaPF (Figure 12B). A significant increase in gas accumulation was observed in all microbiota types except BaBiBl (Figure 12F).

These results suggest that the physical and mental effects of personalized granola consumption vary across gut microbiota types, with common effects on defecation and gut microbiota-dependent effects on mood disturbances.

## 4 Discussion

In this study, we demonstrated that personalized granola, designed to match each individual’s gut microbiota composition, significantly increased metabolites, including SCFAs, and contributed to mood improvement. Furthermore, the specific metabolites that increased following personalized granola consumption varied among the gut microbiota types defined in this study. In a previous study examining intestinal metabolites in 33 healthy men and women who consumed the same fruit granola used as the base for this study, we found that granola intake increased medium-chain fatty acids (having a carbon number of eight or more). However, no significant increase in SCFAs was observed (Yamauchi et al., 2023). In contrast, in the personalized granola experiment, an overall analysis of 93 participants revealed an increasing trend of acetic acid and a significant increase in caproic acid. Additionally, the levels of lactic acid and succinic acid, which are involved in SCFA production, also increased. Furthermore, in a subset analysis of <20 participants, a significant increase in acetic acid, butyric acid, and caproic acid was observed across the different gut microbiota types. Although direct comparisons with previous studies are challenging owing to differences in sample size and consumption duration, the results of this study suggest that personalized granola may be more effective in promoting SCFA production than standard granola.

Additionally, we observed a significant decrease in alpha diversity (Shannon index) when all participants were analyzed together, but no significant changes were detected when participants were stratified by gut microbiota type (Supplementary Figures 2, 3). One likely explanation is the reduced sample size in each microbiota-type subgroup (approximately 20 participants), which may have resulted in insufficient statistical power to detect within-group changes. Moreover, while the overall participants included a broad range of microbial compositions, participants within the same microbiota type likely had more similar baseline microbiota structures, resulting in less variation in alpha diversity metrics over time. These factors combined may explain the discrepancy between the overall and subgroup-level results.

Additionally, questionnaire results from this study indicated a significant increase in stool volume following personalized granola consumption. In contrast, previous studies on granola consumption have primarily reported increased stool frequency rather than stool volume (Yamauchi et al., 2023). Some studies have reported improvements in constipation symptoms among healthy Japanese women and patients undergoing hemodialysis (Higgins and Johanson, 2004). Given that increased dietary fiber intake can lead to both greater stool volume and higher defecation frequency and may help alleviate constipation (Park and Jhon, 2009; Watanabe et al., 2018), the increase in stool volume observed in this study following personalized granola consumption may serve as a potential intervention for constipation relief, similar to previous studies on granola consumption. However, personalized granola consumption was also associated with increased bloating and gas accumulation. Specifically, 56.99% (53 out of 93 participants) reported abdominal bloating, and 68.81% (64 out of 93 participants) reported a sensation of gas accumulation during the intervention period. While indigestible nutrients, such as dietary fiber, can aid in constipation relief, excessive intake has been reported to cause abdominal bloating and gas accumulation (Zhang et al., 2020; Mysonhimer and Holscher, 2022). Most of the prebiotic toppings used in personalized granola contain higher amounts of dietary fiber per gram than standard granola, suggesting that personalized granola may have a greater overall dietary fiber content. Additionally, dietary fiber that is rapidly fermented in the large intestine is sometimes classified as a fermentable dietary fiber (Delcour et al., 2016). Inulin, a common ingredient in personalized granola, is one such fermentable dietary fiber, and *in vitro* studies have shown that inulin fermentation produces more gas compared with that of β-glucan, which is found in oat bran and barley (Carlson et al., 2017). This suggests that the increased fermentation of inulin in the large intestine may contribute to the sensation of abdominal distension. According to the 2023 National Health and Nutrition Survey, the average dietary fiber intake among Japanese individuals was 17.8 g, which falls below the 2025 recommendation of 25 g (Ministry of Health, 2024, 2025). While it remains unclear whether the dietary fiber intake from personalized granola was excessive, it is likely that the increase in fiber consumption contributed to bloating and gas accumulation.

Regarding POMS2, the overall analysis indicated that personalized granola consumption led to an improvement in TMD. Additionally, when examined based on gut microbiota type, improvements were observed across various POMS2 scales. Previous studies have reported that healthy adults with low prebiotic food intake exhibited improvements in TMD scores on POMS2 after an 8-week intervention with high-prebiotic foods (Freijy et al., 2022). These findings align closely with the results of the present study. SCFAs are considered potential role-players through which prebiotics, such as those used in this study, influence mood disturbances. Specifically, butyric acid and propionic acid are believed to act directly on the afferent vagus nerve system, transmitting neural signals to the brain and contributing to stress relief (Goswami et al., 2018; Dalile et al., 2019; La Torre et al., 2021). Moreover, SCFAs have been shown to modulate the reactivity of the hypothalamic-pituitary-adrenal (HPA) axis, attenuating the cortisol response to psychosocial stress (Dalile et al., 2020). Additionally, a meta-analysis investigating the effects of dietary interventions on mental health beyond TMD reported that dietary fiber-rich interventions improved depressive symptoms (Firth et al., 2019). However, despite these findings, research on prebiotic interventions remains limited. Recent systematic reviews indicate insufficient evidence supporting the effectiveness of inulin and galacto-oligosaccharides in improving mental health conditions such as depression, bipolar disorder, and schizophrenia (Ribera et al., 2024). Therefore, further investigation is needed to determine whether prebiotic-containing foods, such as those examined in this study, have a beneficial impact on mood states and mental health disorders.

When analyzing the metabolites produced by each gut microbiota type, we observed that different metabolites increased depending on the gut microbiota type. This variation is considered reasonable, as each microbiota type is characterized by distinct dominant bacterial species, and the metabolites produced vary accordingly. However, one common feature among all microbiota types examined in this study was the presence of *Bacteroides* as a dominant bacterial genus. Previous studies have identified propionate as the main metabolic product of *Bacteroides* (Chambers et al., 2019; Aoki et al., 2021), suggesting that propionate levels should have increased across all microbiota types. Contrary to this expectation, no significant increase in propionate was observed. One possible explanation for this finding is that SCFAs produced in the gut are rapidly absorbed into the bloodstream (Kaji et al., 2015). Additionally, cross-feeding interactions between gut bacteria may play a role. Cross-feeding refers to a symbiotic relationship in which metabolites produced by one bacterial species serve as nutrients for another (Culp and Goodman, 2023). Among SCFAs, acetic acid, butyric acid, and propionic acid are often considered as the final metabolic products (Culp and Goodman, 2023). However, acetic acid can also serve as a precursor, stimulating the growth of butyrate-producing bacteria, thereby promoting butyrate production (Barcenilla et al., 2000; Duncan et al., 2004). Additionally, acetic acid and propionic acid produced by *Veillonella* may be utilized by other symbiotic bacteria to support their growth (Zhang et al., 2024). These findings suggest that increases in specific bacterial species are not solely dictated by direct associations with specific metabolite production. Instead, gut microbiota composition and metabolic output are influenced by complex cross-feeding interactions. Although *Bacteroides* was present in all microbiota types in this study, the metabolites produced varied, likely depending on the presence of other bacterial genera, such as *Blautia*, *Faecalibacterium*, and *Bifidobacterium*. Owing to the highly complex nature of microbial interactions, further studies should focus on a broader range of bacterial species, beyond the six genera used in this study to define personalized granola composition, to better elucidate these relationships. Despite the complexity of metabolite production, an increase in *Bifidobacterium* abundance was observed in most microbiota types examined in this study. Indeed, previous studies have reported that inulin, a prebiotic associated with *Bacteroides* as defined in this study, promotes the growth of *Bifidobacterium* (Costabile et al., 2010; Petry et al., 2012; Birkeland et al., 2020). However, no clear explanation for the discrepancy between bacterial abundance and metabolite production currently exists, that is, while the relative abundance of certain bacterial genera increases, the expected concomitant increase in specific metabolites is not consistently observed.

In this study, we observed a tendency for *Bacteroides* and *Faecalibacterium* to decrease following the intervention (Figures 5A,B). While the exact mechanisms remain unclear, one possible explanation is that the bacterial abundance was expressed in relative terms, and the significant increase in *Bifidobacterium* may have led to an apparent decrease in other genera. In compositional data analysis, such changes do not necessarily indicate an actual decline in bacterial load but rather a shift in the proportion of different taxa within the microbial community. Future studies incorporating absolute quantification methods (e.g., qPCR) are warranted to clarify whether these shifts reflect true reductions or relative effects due to the expansion of other bacterial taxa. Additionally, a previous study has reported that dietary intake of prebiotic-containing granola significantly decreased *Bacteroides* abundance (Sasaki et al., 2025), suggesting that certain prebiotic components in our formulation might have contributed to the observed reduction.

This study has certain limitations. The first limitation concerns the definition used to create personalized granola. In this study, we selected six bacterial genera and their corresponding prebiotic toppings based on previous research. However, the relationship between gut bacteria and prebiotics is not strictly one-to-one. In other words, a single prebiotic topping may stimulate metabolic activity in multiple bacterial species, leading to increased metabolite production. For instance, as discussed earlier, inulin not only increases the relative abundance of *Bifidobacterium* but also affects *Bacteroides* and may contribute to SCFA production (Birkeland et al., 2020). Additionally, in this study, prebiotic toppings were selected based on their compatibility with the granola formulation. However, to further optimize precision nutrition, it may be necessary to explore a broader range of ingredients. In this study, the gut microbiota type was determined based on the top three bacterial genera out of six candidate genera. However, there is no clear rationale for selecting only the top three. Future studies should explore alternative methods for defining gut microbiota types, potentially considering a broader range of bacterial species or different classification criteria. Furthermore, the selection of prebiotic toppings based on genus-level abundance assumes that the identified bacteria will actively metabolize the assigned prebiotic *in vivo*. While this assumption was grounded in prior *in vitro* and *in vivo* studies, the actual functionality and response may vary between individuals. Future studies should consider incorporating functional validation (e.g., metabolite response testing or *in vitro* fermentation assays) to confirm the appropriateness of personalized combinations. Additionally, baseline SCFA levels at the start of the experiment may also play a crucial role. In this study, when analyzing the correlation between initial metabolite levels (week 0) and changes in those metabolites over time (week 8 vs. week 0), a significant negative correlation was observed for most metabolic substances (Supplementary Figure 4). In other words, individuals with higher baseline metabolite levels tended to experience greater increases in metabolite production after consuming granola. Given this finding, pre-experiment metabolite measurement and classification may be necessary. Establishing specific criteria for baseline metabolite levels and stratifying participants accordingly could allow the formulation of tailored granola interventions based on individual metabolic profiles. Moving forward, gut microbiota classification should not rely solely on bacterial composition but should incorporate multiple perspectives, including metabolic activity and other host factors, to refine precision nutrition strategies. The second limitation concerns the study design. This study employed a single-group before-and-after comparison, which has the advantage of minimizing individual differences by comparing participants to themselves. However, this design does not fully account for potential placebo effects and psychological expectations. To address this limitation, future research should implement a randomized, double-blind study with two groups: an intervention group consuming granola containing three prebiotic toppings tailored to their gut microbiota type and a control group consuming granola with three prebiotic toppings that do not match their gut microbiota type. Such a study design would allow for a more rigorous evaluation of whether matching dietary interventions to gut microbiota type is essential for optimizing health outcomes. Furthermore, this study did not include a control group consuming the base granola without any prebiotic toppings. As a result, it is not possible to distinguish whether the observed effects were due to the base granola itself or to the addition of specific prebiotics. Although we previously conducted a separate study in which participants consumed the same base granola without prebiotic toppings, and observed that individuals with a high abundance of *Prevotella 9* showed an increase in SCFA levels (Yamauchi et al., 2023), that study did not allow for the direct comparison between base granola and personalized prebiotic formulations. Therefore, future studies should consider including both a base granola-only control group and a personalized prebiotic group to more rigorously isolate the independent and combined effects of base granola and prebiotic additions on gut microbiota and metabolic outcomes. The third limitation is that dietary intake was not fully controlled. Participants were instructed not to increase their alcohol or probiotic supplement intake beyond their usual levels and to avoid making major dietary changes during the study period. However, their overall diet was not standardized, meaning that individual dietary habits may have influenced the results. In addition, although dietary intake was monitored using the “Calomama” app, participants did not consistently log all meals, and the recorded data showed signs of underreporting. As a result, the calculated values for energy and nutrient intake were considered unreliable and were not included in the final analysis. This lack of accurate dietary data limits our ability to adjust for potential dietary confounders in interpreting the study results. Additionally, 38 out of 93 participants reported consuming probiotic-containing foods (such as yogurt, fermented milk beverages, cheese, and natto) during the study period. Although we did not restrict probiotic food intake, participants were instructed to maintain their usual consumption habits and avoid major dietary changes. Therefore, while the influence of probiotic intake on gut microbiota cannot be entirely ruled out, we believe its impact was limited, especially since baseline microbiota compositions were measured and used as the reference for each participant’s outcome evaluation. To further evaluate the influence of probiotic-containing foods on the increase in *Bifidobacterium*, we conducted a subgroup analysis comparing participants who consumed probiotics during the intervention period (*n* = 38) and those who did not (*n* = 55). As shown in Supplementary Figure 5, a significant increase in *Bifidobacterium* was observed in both groups, indicating that the personalized granola itself likely played a dominant role in promoting *Bifidobacterium* growth. Therefore, while we cannot fully rule out the potential confounding effects of probiotics, these results suggest that their influence was limited in this study. Additionally, while participants were asked to maintain a consistent meal timing, the exact time of food intake was not strictly controlled. Previous studies have shown that the gut microbiota follows a circadian rhythm, which is influenced by meal timing and fasting duration (Thaiss et al., 2014; Sasaki et al., 2019). Thus, a later-than-usual dinner or a shorter fasting period before breakfast could potentially affect gut microbiota composition and SCFA production. Next, unmeasured and uncontrolled confounding factors may have impacted the study outcomes. Gut microbiota composition and SCFA production are influenced by multiple variables, including exercise, sleep, and social factors such as work-related stress and marital status. These factors should be taken into account when interpreting the results, as they may introduce potential biases. Finally, we note that psychological and bowel-related outcomes were assessed using self-administered questionnaires, including the AIS and POMS2. Although these instruments are validated, self-reported data are inherently subject to potential biases such as recall bias, reporting inaccuracy, or social desirability bias. While participants were instructed to respond as truthfully as possible and were assured of confidentiality, these biases cannot be entirely ruled out and should be considered when interpreting the results.

In this study, gut microbiota types were classified based on gut microbiota composition, and participants consumed personalized granola containing prebiotics tailored to their bacterial type. As a result, different microbiota types exhibited distinct metabolite production patterns, and a significant increase in *Bifidobacterium* abundance was observed. Additionally, participants reported a significant increase in stool volume, and total mood disturbance (TMD) scores from the POMS2 questionnaire significantly improved, indicating beneficial effects on both bowel function and mental health. In terms of microbial metabolites, levels of caproic acid and formic acid showed significant changes over the eight-week intervention. These findings suggest that personalized granola, formulated according to individual gut microbiota types, can effectively promote beneficial bacterial growth, enhance metabolite production, and improve physiological and psychological outcomes.

## Data Availability

The datasets presented in this study can be found in the Figshare repository at the following link: https://dx.doi.org/10.6084/m9.figshare.29554772.
