# Supplementary material for: Effectiveness of personalized granola tailored to the gut microbiota for improving gut environment and mood states
Source: Front Microbiol. 2025 Jul 25;16:1607918. doi: 10.3389/fmicb.2025.1607918 (PMC12332753; doi:10.3389/fmicb.2025.1607918)
Supplement: Supplementary file 1 [file Presentation_1.pdf]

Supplementary Material

1 Supplementary Table

1.1 Supplemental Table 1. Ingredient composition of Fruit granola and each prebiotic-toppings.

Fruit granola	Inulin	Barley	FOS	RS	GOS	Hi-Cacao
Oats, dried fruit (papaya, raisins, apples, strawberries), rye flour, sugar, wheat flour, coconut, dextrin, vegetable oil, rice flour, corn flour, soluble dietary fiber, pumpkin seeds, almond powder, salt, wheat bran , brown rice flour, fructooligosaccharide syrup, lactose / glycerin, sodium citrate, acidulant, antioxidants	Oats, rice puffs, inulin, wheat puffs (wheat flour, starch, malt extract, salt), sugar, dextrin, almond powder, salt / flavoring, leavening agent, antioxidant (vitamin E)	Oats, rice puffs, barley, wheat puffs (wheat flour, starch, malt extract, salt), dextrin, sugar, almond powder, salt / leavening agent, antioxidant (vitamin E)	Oats, wheat puffs (wheat flour, starch, malt extract, salt), fructo-oligosaccharides, wheat flour, dextrin, rice flour, corn flour, rye flour, almond powder, salt, brown rice flour / flavoring, leavening agent, antioxidant (vitamin E)	Resistant starch, wheat flour, sugar, vegetable oil, rice flour, corn flour, rye flour, brown rice flour, salt, dextrin / flavoring, safflower yellow, antioxidant (vitamin E)	Rice puffs, galacto-oligosaccharide (contains milk ingredients), oats, wheat flour, dextrin, rice flour, corn flour, rye flour, almond powder, salt, brown rice flour / flavoring, antioxidant (vitamin E)	Wheat flour, sugar, oats, chocolate (contains milk ingredients), wheat puffs (wheat flour, starch, malt extract, salt), rice flour, corn flour, cacao mass, rye flour, almond powder, dextrin, brown rice flour, salt / emulsifier (soybean derived), leavening agent, antioxidant (vitamin E), flavoring

(vitamin E, rosemary extract), modified starch, niacin, calcium pantothenate, vitamin A, vitamin B6, vitamin B1, folic acid, vitamin D, vitamin B12

FOS, fructooligosaccharide; RS, resistant starch; GOS, galactooligosaccharide; Hi-Cacao, high cacao chocolate.

1.2 Supplemental Table 2. Summary of PERMANOVA results for gut microbiota beta-diversity across groups

adj p-value		BaBiBl			BaBiF			BaFBl			BaPF			BaRF		
		0wk	4wk	8wk	0wk	4wk	8wk	0wk	4wk	8wk	0wk	4wk	8wk	0wk	4wk	8wk
BaBiBl	0wk	-	0.9999	0.3080	0.0156*	0.0018*	0.0018*	0.0018*	0.1360	0.1399	0.0018*	0.0018*	0.0018*	0.0018*	0.0048*	0.0018*
	4wk		-	0.9999	0.0500*	0.0018*	0.0048*	0.0018*	0.0188*	0.0188*	0.0018*	0.0018*	0.0018*	0.0033*	0.0078*	0.0033*
	8wk			-	0.07080	0.0121*	0.0358*	0.0018*	0.0121*	0.0018*	0.0018*	0.0018*	0.0018*	0.0018*	0.0033*	0.0018*
BaBiF	0wk				-	0.4562	0.8400	0.0018*	0.0156*	0.0063*	0.0018*	0.0018*	0.0018*	0.0518	0.2730	0.2217
	4wk					-	0.9999	0.0018*	0.0018*	0.0018*	0.0018*	0.0018*	0.0018*	0.0018*	0.0156*	0.0108*
	8wk						-	0.0018*	0.0033*	0.0018*	0.0018*	0.0018*	0.0018*	0.0018*	0.0358*	0.0212*
BaFBl	0wk							-	0.9999	0.9999	0.0018*	0.0018*	0.0018*	0.0179*	0.0018*	0.0018*
	4wk								-	0.9999	0.0018*	0.0018*	0.0018*	0.0392*	0.0179*	0.0156*
	8wk									-	0.0018*	0.0018*	0.0018*	0.0275*	0.015*	0.0048*
BaPF	0wk										-	0.9999	0.9340	0.0018*	0.0018*	0.0018*
	4wk											-	0.9999	0.0018*	0.0018*	0.0018*
	8wk												-	0.0018*	0.0018*	0.0018*
BaRF	0wk													-	0.8400	0.7767
	4wk														-	0.9999
	8wk															-

* adjusted p-value<0.05; evaluated using the PERMANOVA

2 Supplementary Figures legends

2.1 Supplemental Figure 1. Compare the overall composition of gut microbiota by week and type

Comparison of bacterial beta diversity between weeks and type of gut microbiota. Principal coordinate analysis (PCoA) plots of weighted UniFrac distance metrics obtained from sequencing the 16s rDNA gene in fecal samples.

2.2 Supplemental Figure 2. Personalized granola consumption reduces gut microbiota diversity.

Bacterial alpha diversity in all subjects. (A) Chao1, (B) Shannon index, (C) Simpson index. All value are represented as mean ± SEM (n=93). ## p<0.01, # p<0.05, evaluated using the Friedman test with Dunn's post-hoc test.

2.3 Supplemental Figure 3. Personalized granola consumption reduces gut microbiota diversity in any type of gut microbiota.

Bacterial alpha diversity by each type of gut microbiota. (A) Chao1, (B) Shannon index, (C) Simpson index. All value are represented as mean ± SEM (BaBiBl type: n=19, BaBiF type: n=20, BaFBl type: n=18, BaPF type: n=19, BaRF type: n=17). ## p<0.01, # p<0.05, evaluated using the Friedman test with Dunn's post-hoc test to compare between weeks within the gut microbiota type.

2.4 Supplemental Figure 4. Correlation Analysis of Gut Microbiota Metabolites Before and After the Experiment.

Correlation analysis of gut microbiota metabolites. (A) Acetic acid, (B) Propionic acid, (C) Isobutyric acid, (D) Butyric acid, (E) Isovaleric acid, (F) Valeric acid, (G) Caproic acid, (H) Formic acid, (I) Lactic acid, (J) Malonic acid, (K) Succinic acid, (L) 2-Methylvaleric acid, and (M) 4-Methylvaleric acid. The numbers in the diagram are the r-value and p-value calculated using Spearman's rank correlation coefficient.

2.5 Supplemental Figure 5. Consumption of personalized granola increased the relative abundance of *Bifidobacterium*, independent of probiotic intake during the intervention period.

The relative abundance of *Bifidobacterium* in (A) probiotic intake group (n=38) and (B) no probiotic intake group (n=55) during the intervention period. All values are represented as mean ± SEM. ## p<0.01, evaluated using the Friedman test with Dunn's post-hoc test.
