# Supplementary material for: Effectiveness of personalized granola tailored to the gut microbiota for improving gut environment and mood states
Source: Front Microbiol. 2025 Jul 25;16:1607918. doi: 10.3389/fmicb.2025.1607918 (PMC12332753; doi:10.3389/fmicb.2025.1607918)
Supplement: Supplementary file 2 [file Presentation_2.pptx]

## Slide 1
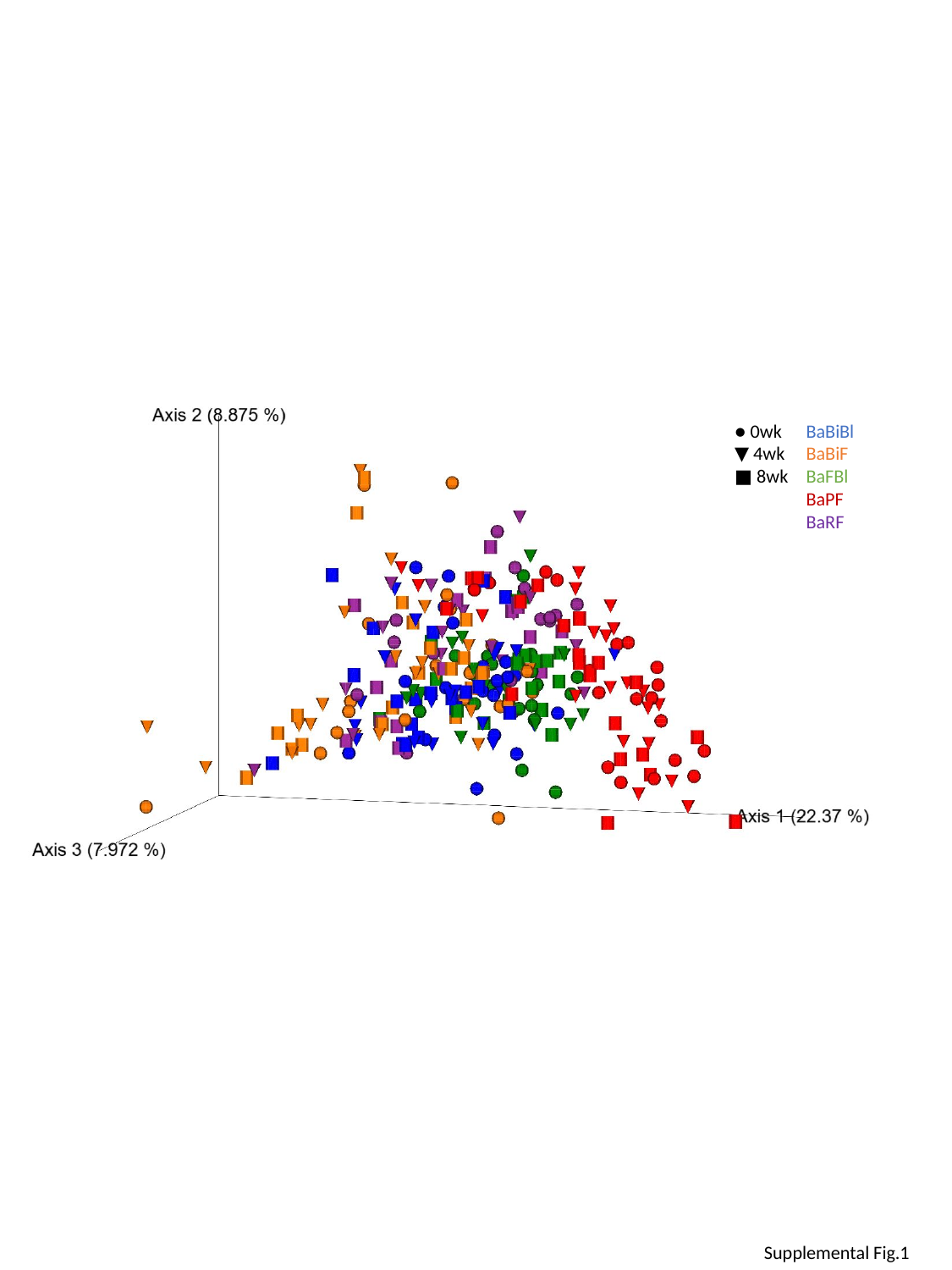

● 0wk
▼ 4wk
■ 8wk
BaBiBl
BaBiF
BaFBl
BaPF
BaRF
Supplemental Fig.1

## Slide 2
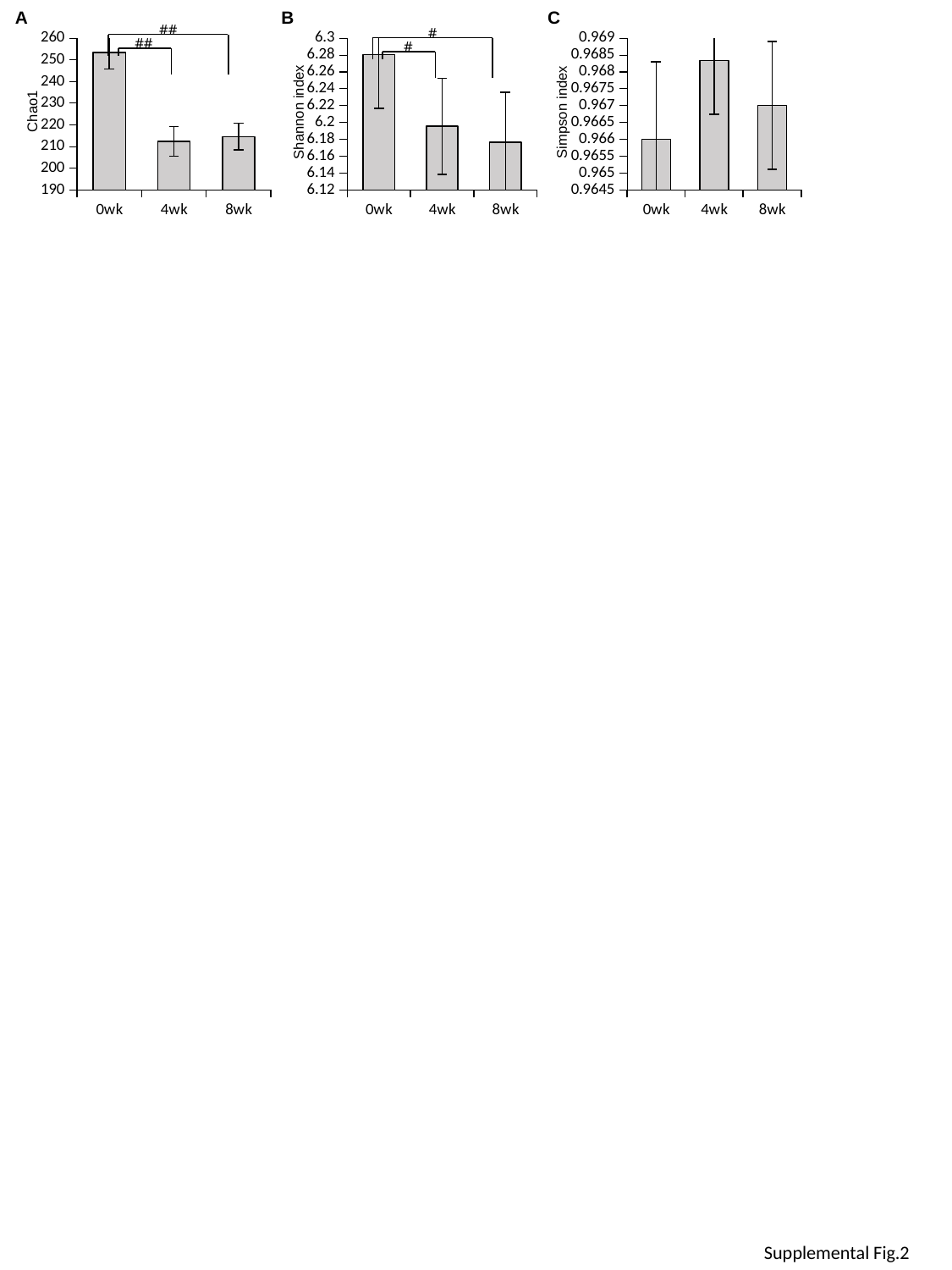

A
B
C
##
#
### Chart
| Category | chao1 |
|---|---|
| 0wk | 253.48049526313898 |
| 4wk | 212.33782998742325 |
| 8wk | 214.61403806967525 |
### Chart
| Category | shannon |
|---|---|
| 0wk | 6.280389058903194 |
| 4wk | 6.195496426438167 |
| 8wk | 6.176663617791733 |
### Chart
| Category | simpson |
|---|---|
| 0wk | 0.966000304771392 |
| 4wk | 0.9683428012904279 |
| 8wk | 0.9670035459053533 |##
#
Chao1
Shannon index
Simpson index
Supplemental Fig.2

## Slide 3
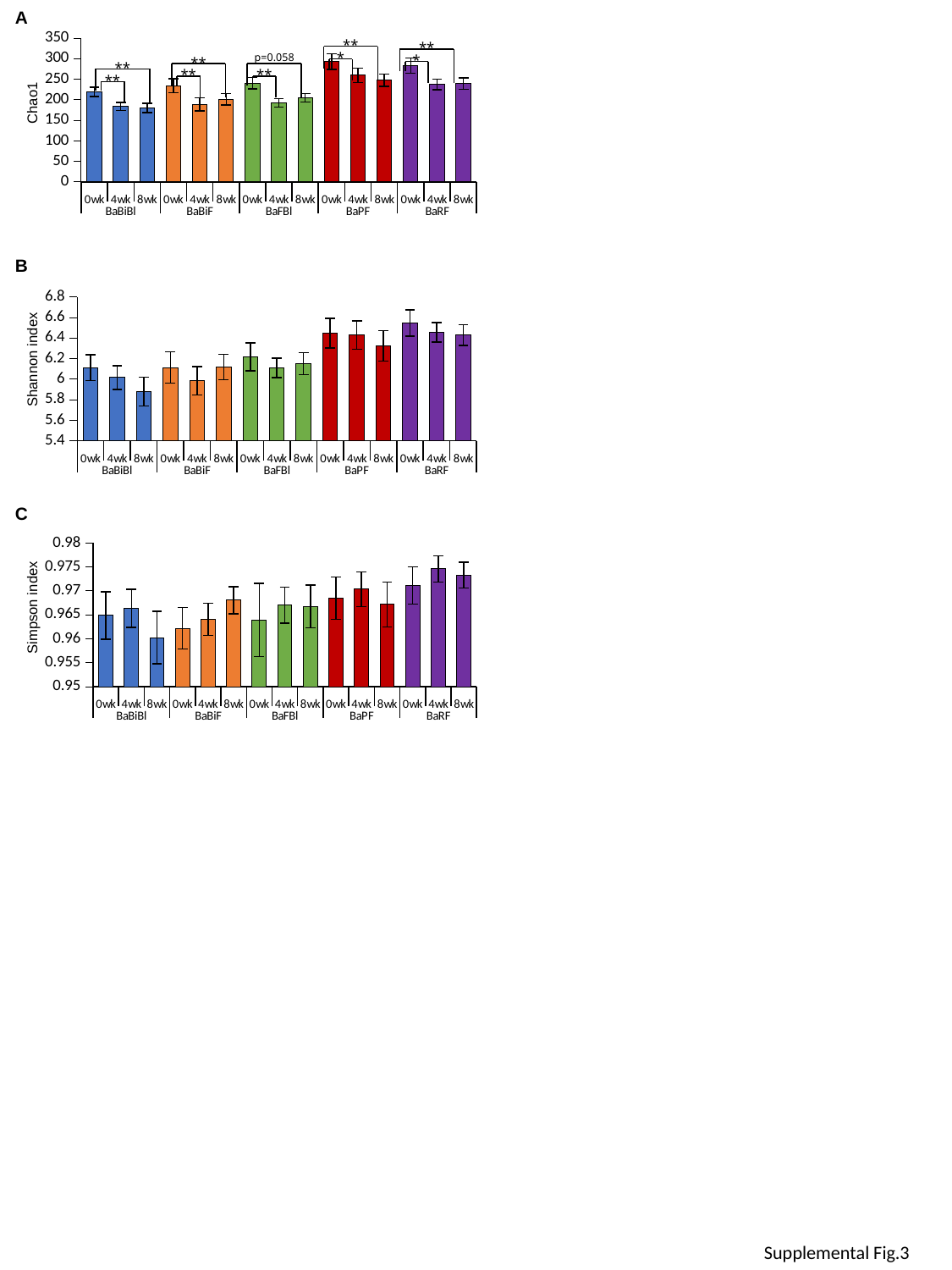

A
### Chart
| Category | chao1 |
|---|---|
| 0wk | 219.78763575605674 |
| 4wk | 184.11453634085214 |
| 8wk | 180.21798245614036 |
| 0wk | 234.4045346320347 |
| 4wk | 188.84166666666664 |
| 8wk | 201.41811507936504 |
| 0wk | 240.5255952380952 |
| 4wk | 192.250815696649 |
| 8wk | 205.38742283950614 |
| 0wk | 292.7875517598343 |
| 4wk | 259.6179505135388 |
| 8wk | 247.5616436100132 |
| 0wk | 283.36506410256413 |
| 4wk | 237.16898836457656 |
| 8wk | 239.58877217553686 |**
**
*
p=0.058
*
**
**
**
**
**
Chao1
B
### Chart
| Category | shannon |
|---|---|
| 0wk | 6.110885559051345 |
| 4wk | 6.016396114046542 |
| 8wk | 5.879370514222112 |
| 0wk | 6.112747697542574 |
| 4wk | 5.985334561464061 |
| 8wk | 6.118697880232805 |
| 0wk | 6.216113528908507 |
| 4wk | 6.110358894499635 |
| 8wk | 6.153149275688154 |
| 0wk | 6.449419674089849 |
| 4wk | 6.430636132554772 |
| 8wk | 6.324136449360497 |
| 0wk | 6.546199151594675 |
| 4wk | 6.456427290407862 |
| 8wk | 6.428527455761769 |Shannon index
C
### Chart
| Category | simpson |
|---|---|
| 0wk | 0.9648739593240198 |
| 4wk | 0.9663669243500701 |
| 8wk | 0.9602621976344253 |
| 0wk | 0.96220353548104 |
| 4wk | 0.9640774173855824 |
| 8wk | 0.9680688935293151 |
| 0wk | 0.9639561128534252 |
| 4wk | 0.9670160477478008 |
| 8wk | 0.966783874308172 |
| 0wk | 0.9684687138619302 |
| 4wk | 0.9703760215717738 |
| 8wk | 0.9671789069276955 |
| 0wk | 0.9711315771896435 |
| 4wk | 0.9745820070612566 |
| 8wk | 0.9733109301977543 |Simpson index
Supplemental Fig.3

## Slide 4
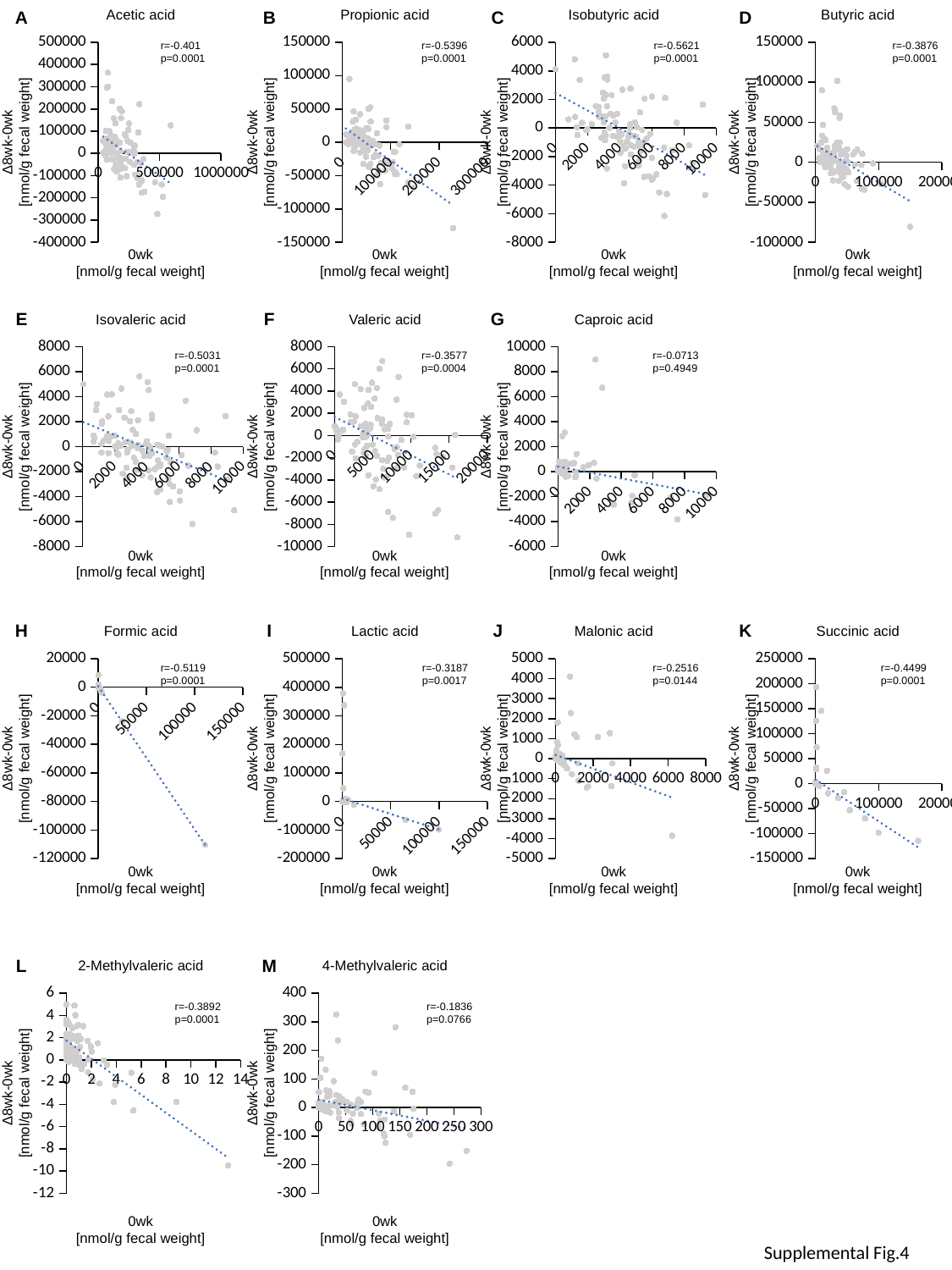

Acetic acid
Propionic acid
Isobutyric acid
Butyric acid
A
B
C
D
### Chart
| Category | Acetic acid |
|---|---|
### Chart
| Category | Propionic acid |
|---|---|
### Chart
| Category | Isobutyric acid |
|---|---|
### Chart
| Category | Butyric acid |
|---|---|r=-0.401
p=0.0001
r=-0.5396
p=0.0001
r=-0.5621
p=0.0001
r=-0.3876
p=0.0001
Δ8wk-0wk
[nmol/g fecal weight]
Δ8wk-0wk
[nmol/g fecal weight]
Δ8wk-0wk
[nmol/g fecal weight]
Δ8wk-0wk
[nmol/g fecal weight]
0wk
[nmol/g fecal weight]
0wk
[nmol/g fecal weight]
0wk
[nmol/g fecal weight]
0wk
[nmol/g fecal weight]
E
F
G
Isovaleric acid
Valeric acid
Caproic acid
### Chart
| Category | Isovaleric acid |
|---|---|
### Chart
| Category | Valeric acid |
|---|---|
### Chart
| Category | Caproic acid |
|---|---|r=-0.5031
p=0.0001
r=-0.3577
p=0.0004
r=-0.0713
p=0.4949
Δ8wk-0wk
[nmol/g fecal weight]
Δ8wk-0wk
[nmol/g fecal weight]
Δ8wk-0wk
[nmol/g fecal weight]
0wk
[nmol/g fecal weight]
0wk
[nmol/g fecal weight]
0wk
[nmol/g fecal weight]
H
I
J
K
Formic acid
Lactic acid
Malonic acid
Succinic acid
### Chart
| Category | Formic acid |
|---|---|
### Chart
| Category | Lactic acid |
|---|---|
### Chart
| Category | Malonic acid |
|---|---|
### Chart
| Category | Succinic acid |
|---|---|r=-0.5119
p=0.0001
r=-0.3187
p=0.0017
r=-0.2516
p=0.0144
r=-0.4499
p=0.0001
Δ8wk-0wk
[nmol/g fecal weight]
Δ8wk-0wk
[nmol/g fecal weight]
Δ8wk-0wk
[nmol/g fecal weight]
Δ8wk-0wk
[nmol/g fecal weight]
0wk
[nmol/g fecal weight]
0wk
[nmol/g fecal weight]
0wk
[nmol/g fecal weight]
0wk
[nmol/g fecal weight]
L
M
2-Methylvaleric acid
4-Methylvaleric acid
### Chart
| Category | 2-Methylvaleric acid |
|---|---|
### Chart
| Category | 4-Methylvaleric acid |
|---|---|r=-0.3892
p=0.0001
r=-0.1836
p=0.0766
Δ8wk-0wk
[nmol/g fecal weight]
Δ8wk-0wk
[nmol/g fecal weight]
0wk
[nmol/g fecal weight]
0wk
[nmol/g fecal weight]
Supplemental Fig.4

## Slide 5
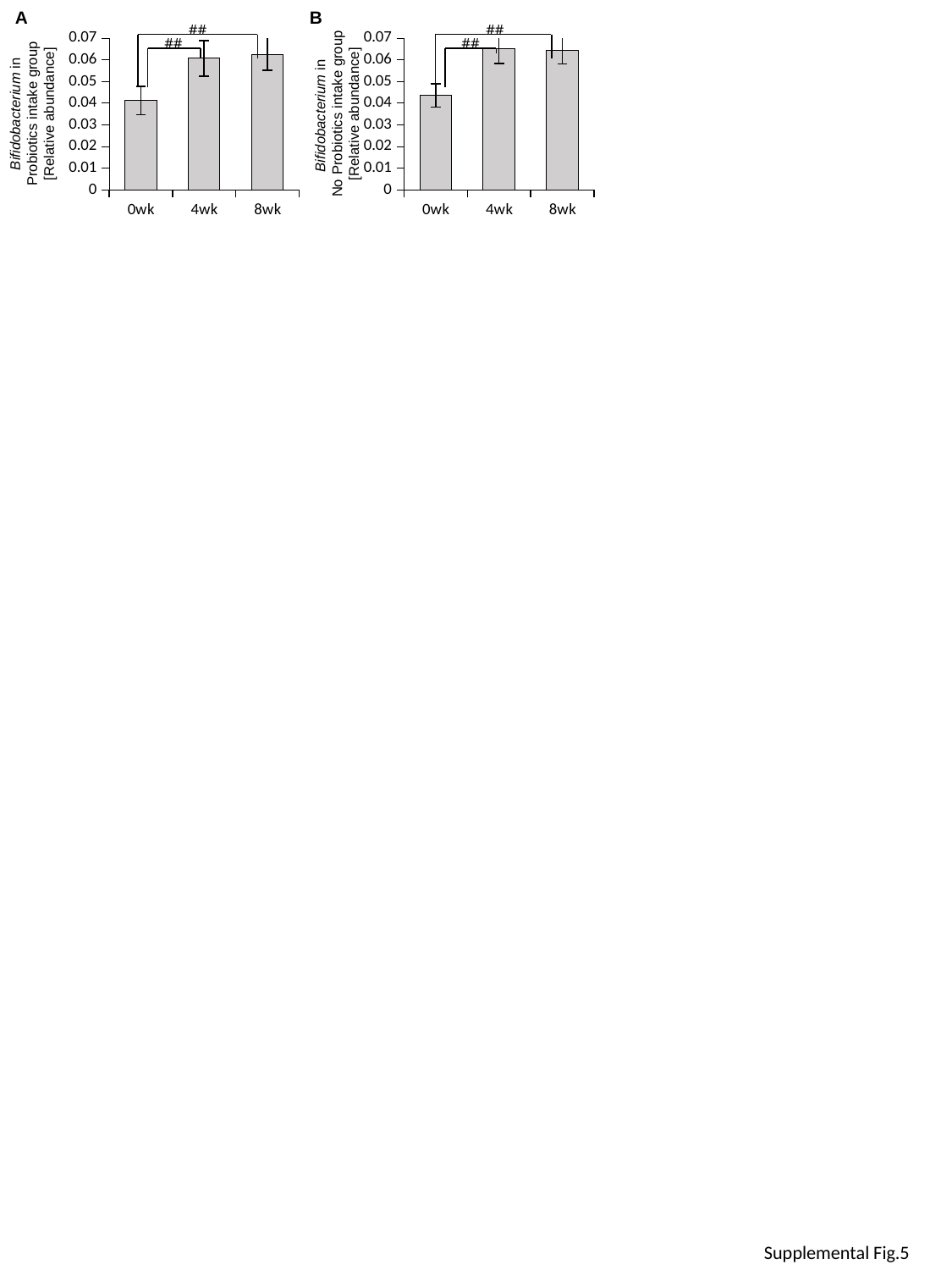

A
B
##
##
### Chart
| Category | Probiotics |
|---|---|
| 0wk | 0.041292217629026996 |
| 4wk | 0.06070959592116606 |
| 8wk | 0.06263233282945863 |
### Chart
| Category | None |
|---|---|
| 0wk | 0.04364589926718569 |
| 4wk | 0.06509922165987066 |
| 8wk | 0.06430182820577904 |##
##
Bifidobacterium in Probiotics intake group
[Relative abundance]
Bifidobacterium in
No Probiotics intake group
[Relative abundance]
Supplemental Fig.5
